# Regulation with cell size ensures mitochondrial DNA homeostasis during cell growth

**DOI:** 10.1038/s41594-023-01091-8

**Published:** 2023-09-07

**Authors:** Anika Seel, Francesco Padovani, Moritz Mayer, Alissa Finster, Daniela Bureik, Felix Thoma, Christof Osman, Till Klecker, Kurt M. Schmoller

**Affiliations:** 1 Institute of Functional Epigenetics, Molecular Targets and Therapeutics Center, Helmholtz Zentrum München, Neuherberg, Germany; 2 Institute of Cell Biology, University of Bayreuth, Bayreuth, Germany; 3 Faculty of Biology, Ludwig-Maximilians-Universität München, Planegg-Martinsried, Germany

**Keywords:** DNA replication, Mitochondria, Cell growth, Cellular imaging

## Abstract

To maintain stable DNA concentrations, proliferating cells need to coordinate DNA replication with cell growth. For nuclear DNA, eukaryotic cells achieve this by coupling DNA replication to cell-cycle progression, ensuring that DNA is doubled exactly once per cell cycle. By contrast, mitochondrial DNA replication is typically not strictly coupled to the cell cycle, leaving the open question of how cells maintain the correct amount of mitochondrial DNA during cell growth. Here, we show that in budding yeast, mitochondrial DNA copy number increases with cell volume, both in asynchronously cycling populations and during G1 arrest. Our findings suggest that cell-volume-dependent mitochondrial DNA maintenance is achieved through nuclear-encoded limiting factors, including the mitochondrial DNA polymerase Mip1 and the packaging factor Abf2, whose amount increases in proportion to cell volume. By directly linking mitochondrial DNA maintenance to nuclear protein synthesis and thus cell growth, constant mitochondrial DNA concentrations can be robustly maintained without a need for cell-cycle-dependent regulation.

## Abstract (web summary)

Mitochondrial DNA is critical for cell function, but how growing cells maintain stable concentrations is unclear. Seel et al. find that cells couple mitochondrial DNA copy number directly to cell volume through nuclear-encoded limiting factors, whose amount increases with cell volume.

## Main

As cells grow during the cell cycle, they need to double their DNA content so that each daughter cell obtains the appropriate amount. In fact, a major task of the eukaryotic cell cycle is ensuring that nuclear DNA is replicated once—and only once—during S phase, and extensive research has given us a detailed understanding of this process^1,2^. By contrast, how this is achieved for mitochondrial DNA (mtDNA) is largely unclear.

In many organisms, including humans and yeasts, mtDNA encodes proteins that are essential for oxidative phosphorylation, and cells typically contain many mtDNA copies^3,4^. mtDNA is organized in ‘nucleoids,’ nucleoprotein complexes that can contain one or several copies of mtDNA^5,6^. Although several regulators of mtDNA copy number have been identified^6^, including the nucleoid protein TFAM^7^ and its homolog Abf2 in yeast^8^, mtDNA polymerase^9^, and helicases^10,11^, how cells maintain the correct number of mtDNA copies throughout cell growth is unknown. In contrast to replication of nuclear DNA, mtDNA replication is not strictly coupled to cell-cycle progression. Although some studies have reported cell-cycle-dependent modulation of mtDNA replication rates for human cells^12–14^, mtDNA replication occurs throughout the cell cycle and even continues during long cell-cycle arrests^15–18^. However, if mtDNA replication is not controlled by cell-cycle progression, how can cells then coordinate the amount of mtDNA produced with cell growth?

One possibility is that mitochondrial homeostasis is directly linked to cell size. Indeed, it has been shown that the amount of mitochondria in budding yeast^19^, HeLa^20^, mouse liver^21^, Jurkat and *Drosophila* Kc167 cells^22^ increases roughly in proportion to cell volume. In addition, the number of nucleoids in budding yeast correlates with mitochondrial network volume^23^, and nucleoid number in fission yeast increases with increasing cell volume^24^—suggesting that mtDNA copy number might be linked to cell volume. However, direct evidence for a role for cell volume in mtDNA homeostasis is missing.

Here we show that, in budding yeast, the number of mtDNA copies and nucleoids increases in direct proportion to cell volume. We find that mtDNA maintenance is limited by nuclear-encoded proteins whose abundance increases with cell volume. Supported by mathematical modeling, our results suggest that the overall increase of cellular protein synthesis with increasing cell volume couples mtDNA copy number to cell volume, achieving robust mtDNA homeostasis during cell growth and cell-cycle progression.

## Results

### mtDNA copy number increases with cell volume

**Fig. 1:**
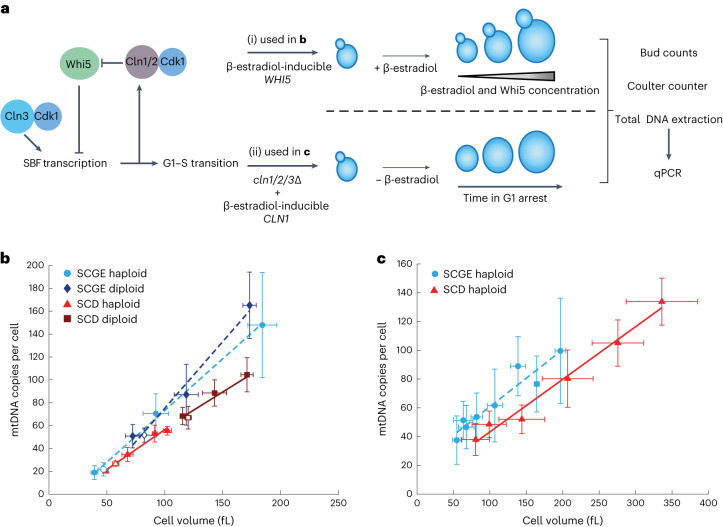
mtDNA increases with cell volume and is modulated by nutrients. **a**, Cell volume was manipulated using two genetic approaches. i, Whi5 concentration was controlled by a β-estradiol-inducible promoter. Higher β-estradiol concentrations led to a prolonged G1 phase, resulting in larger cell volumes in asynchronous steady-state populations. ii, A strain with *cln1*, *cln2*, and *cln3* deletion *(cln1/2/3*∆), in which Cln1 was expressed using a β-estradiol-inducible promoter, was used^29^. In this strain, β-estradiol is necessary for cell proliferation; its removal leads to G1 arrest and continuously increasing cell volumes. **b**, mtDNA copy number as a function of cell volume in asynchronous steady-state populations. Haploid and diploid wild-type (open symbols) and Whi5-inducible strains (filled symbols) in the absence of β-estradiol or in the presence of different β-estradiol concentrations were grown on SCGE (dashed line) or SCD (solid line) medium. After total DNA extraction, mtDNA copy number was determined by measuring the relative concentrations of mtDNA and nuclear DNA. mtDNA concentration was normalized to nuclear DNA concentration, and the budding index was used to estimate the number of nuclear DNA copies per cell. Mean cell volumes of cell populations were measured with a Coulter counter. *n*_Haploid SCGE_ = 4; *n*_diploid SCGE_ = 4; *n*_haploid SCD_ = 3; *n*_diploid SCD_ = 4 biological replicates. **c**, mtDNA copy numbers as a function of cell volume during G1 arrest. Cells were arrested in G1 and collected every hour for 6 h (SCD) or 8 h (SCGE), starting directly after β-estradiol removal. mtDNA copy numbers were measured with qPCR, and cell volumes were measured with a Coulter counter. Lines show linear fits to the means of *n* = 3 biological replicates. Error bars indicate the s.d. Source data

**Extended Data Fig. 1:**
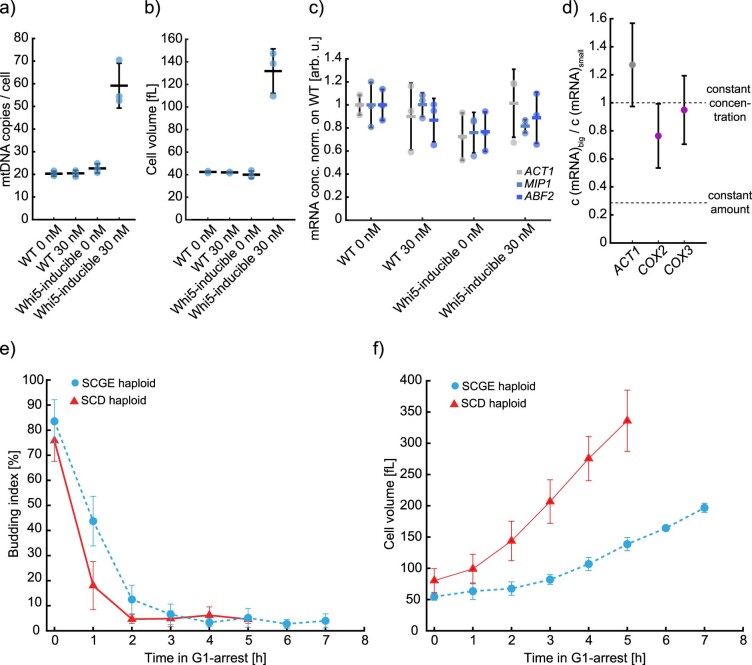
Validation of Whi5 overexpression system, size dependence of transcripts encoded by mtDNA and budding index and cell volume during G1-arrests corresponding to the experiments shown in Fig. 1c. Haploid wild-type (WT) and Whi5-inducible strains were grown on SCGE and treated with β-estradiol as indicated (a-d). **a)** mtDNA copy number was determined by DNA-qPCR. No effect of β-estradiol was found for wild-type cells, whereas the Whi5-inducible strain shows a 3-fold increase. **b)** Mean cell volume was measured with a Coulter counter. As for mtDNA, addition of 30 nM β-estradiol causes no changes in cell volume for wild-type cells. **c)** RT-qPCR was performed to measure transcript levels in wild-type. Cq-values were normalized on *RDN18* and mRNA concentrations were normalized on wild-type levels at 0 nM. No significant changes in transcript levels were found after addition of 30 nM β-estradiol for wild-type cells. Error bars indicate the standard deviation of n = 3 biological replicates (a-c). **d)** mRNA amounts of mitochondrial encoded genes increase with cell volume. mRNA concentrations for *COX2* and *COX3* from same samples as in c were normalized on *RDN18* and the ratio of concentrations in big (30 nM) and small (0 nM) haploid cell populations were calculated. Data points indicate mean of n = 3 biological replicates with error bars indicating the propagated standard error. Cell volumes were measured with a Coulter counter to estimate the concentration ratio expected for an mRNA that is maintained at constant amount. **e)** Budding index (fraction of budding cells) was determined by bud counting. **f)** Cell volume was measured with a Coulter counter. After 2 h most cells are arrested in G1, and mean cell volume continuously increases. To account for the slower growth of cells on SCGE, growth was followed for two additional time points. Shown is the mean of n = 3 biological replicates. Error bars depict standard deviations (e-f). Source data

To understand the role of cell size in the regulation of mtDNA, we first measured the dependence of mtDNA copy number on cell volume in budding yeast. We used haploid and diploid strains carrying the cell-size regulator *WHI5* under the control of a β-estradiol-inducible promoter^25^. Whi5 modulates G1 duration by inhibiting the G1–S transcription factor SBF. Overexpression of Whi5 by addition of β-estradiol therefore initially causes a prolonged G1 phase and increased cell volume. Eventually, cells reach a cell volume that is large enough for division^26^, and after 24 h of growth in the exponential phase, this results in a steady state of asynchronous cell populations with increased mean cell volumes^27,28^ (Fig. 1a). We grew cells on synthetic complete medium with 2% glycerol and 1% ethanol as a non-fermentable carbon source (SCGE). Using β-estradiol concentrations ranging from 0 to 30 nM (haploid) or 60 nM (diploid) and wild-type strains without inducible Whi5, we obtained steady-state cultures with a more than fourfold range in mean cell volume (Extended Data Fig. 1), but with similar doubling times and only moderately shifted cell-cycle fractions^27^. For each culture, we measured cell volume using a Coulter counter, determined bud fractions, purified DNA, and performed quantitative PCR (qPCR) measurements on nuclear and mtDNA to determine the average number of mtDNA copies per cell. For both haploid and diploid strains, we found that mtDNA copy number increases roughly in direct proportion with cell volume (Fig. 1b).

We next asked whether this increase in the amount of mtDNA with increased cell volume is specific to non-fermentable media, in which functional mtDNA is essential. We repeated the experiments using synthetic complete medium with 2% glucose (SCD), in which mtDNA is not essential. Again, we found that the amount of mtDNA increases with cell volume, but at a given cell volume, cells grown on SCD have less mtDNA than do cells grown on SCGE (Fig. 1b).

To test whether the increase of mtDNA is linked to the increase in cell volume, rather than to Whi5 overexpression, we sought an alternative approach to control cell volume. We used a haploid strain in which all three endogenous G1 cyclins (*CLN1*, *CLN2*, and *CLN3*) were deleted, and which is kept alive by a copy of *CLN1* whose expression can be induced by β-estradiol^29^. Upon removal of β-estradiol, the cells arrest in G1 while continuously growing (Extended Data Fig. 1e,f). We collected cells at different time points during G1 arrest and measured cell volume, bud fractions, and mtDNA copy number (Fig. 1a). In accordance with mtDNA being replicated also in G1 (refs. ^15,17^) and confirming the results of the Whi5-inducible system, we found that, as cells grow during G1, mtDNA copy number continuously increases (Fig. 1c). mtDNA copy number is lower in cells grown on SCD than in those grown on SCGE.

### Nucleoid number increases with cell volume

**Fig. 2:**
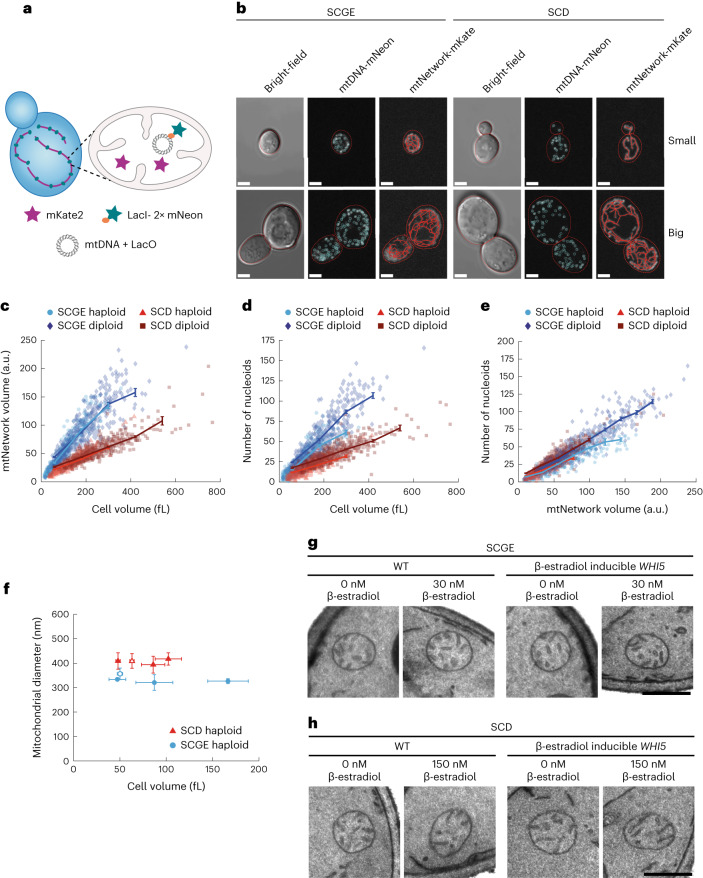
The number of nucleoids and mitochondrial network volume increase with cell volume. **a**, LacI-2×mNeon expressed by nuclear DNA and targeted to the mitochondrial matrix binds LacO repeats integrated into mtDNA. The mitochondrial matrix was visualized with mitochondrially targeted mKate2. **b**, Representative bright-field and confocal live-cell images (maximum intensity projections) of Whi5-inducible diploid cells without (small) or with 60 nM (SCGE) or 150 nM (SCD) β-estradiol (big) are shown, with cell (red dashed lines) and mitochondrial-network (mtNetwork) segmentations and identified mtDNA foci. The skeletonization of the segmentation was used only for visual representation. Corresponding images without network segmentation and nucleoid detection are shown in Extended Data Figure 2a. Scale bars (**b**), 2 µm. **c**, Mitochondrial network volume as a function of cell volume for Whi5-inducible cells grown on SCGE or SCD with different β-estradiol concentrations. **d**, The number of nucleoids per cell as a function of cell volume, for the same cells as in **c**. **e**, The number of nucleoids as a function of mitochondrial network volume, for the same cells as in **c** and **d**. In **b**–**e**, image analysis was performed in 3D. For each condition, 3 biological replicates with 50 images each were analyzed (*n*_haploid SCGE_ = 457; *n*_diploid SCGE_ = 465; *n*_haploid SCD_ = 616; *n*_diploid SCD_ = 606 total cells). Lines connect binned means (shown at the center of the respective bin), with error bars indicating s.e. a.u., arbitrary units. **f**–**h**, Haploid wild-type (open symbols) and Whi5-inducible cells (filled symbols) were grown in SCD or SCGE containing different concentrations of β-estradiol, chemically fixed, and analyzed by transmission electron microscopy. **f**, Mitochondrial diameter as a function of cell volume. For each sample, the diameters of 100 mitochondria were measured from electron micrographs, and cell volumes were calculated from differential interference contrast microscopy (DIC) images taken of the same cultures before chemical fixation. Shown is the mean of the means of *n* = 3 independent experiments; error bars indicate the s.d. of the mean. Cells grown in SCD and those grown in SCGE were analyzed in separate experiments (also see Extended Data Fig. 3a-d). **g**,**h**, Representative electron micrographs of mitochondria of wild-type (WT) and Whi5-inducible cells grown either in SCGE (**g**) or SCD (**h**) containing the indicated concentrations of β-estradiol. Scale bars (**g**,**h**), 500 nm. Additional images are shown in Extended Data Figure 3e,f. Source data

**Extended Data Fig. 2:**
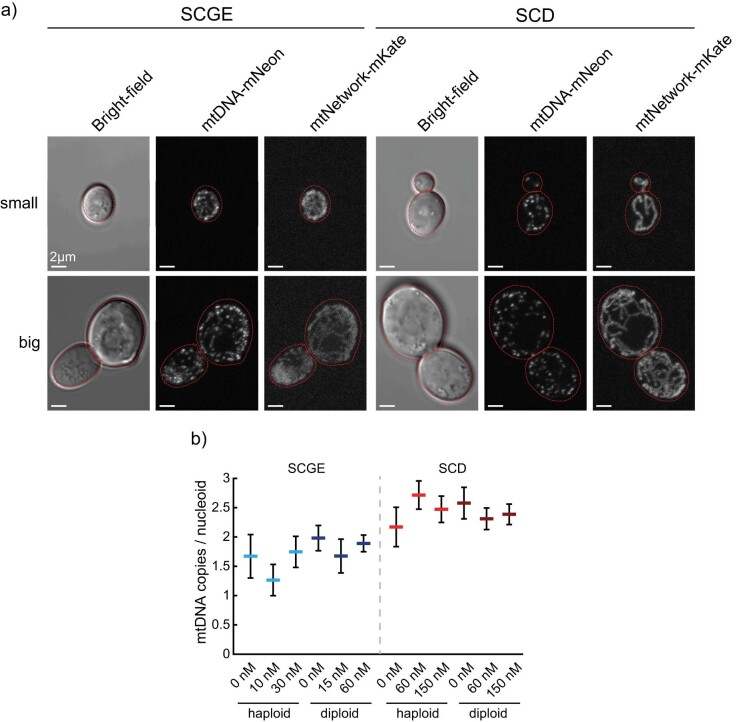
Microscopy images and quantification of mtDNA copies per nucleoid. **a)** Same images as in Fig. 2b are shown without labeling of network segmentation and identified mtDNA. **b)** mtDNA copies per nucleoid for Whi5-inducible haploid and diploid strains. To obtain the presented data, the average mtDNA copy number per cell from Fig. 1b was normalized on cell volume (n_Haploid SCGE_ = 4; n_diploid SCGE_ = 4; n_haploid SCD_ = 3; n_diploid SCD_ = 4) and divided by the average number of nucleoids per cell volume (measured by the Coulter counter) from Fig. 2d (n = 3). Error bars indicate propagated standard errors. The observation that on SCD nucleoids contain on average more mtDNA copies than on SCGE supports previous work that reported a dependence on culture conditions^65^. Source data

mtDNA is organized in nucleoids, which are distributed throughout the mitochondrial network^23^. In principle, the increase in mtDNA with cell volume could be due to an increased number of nucleoids or an increased number of mtDNA copies per nucleoid. To distinguish between the two scenarios, we adapted a previously established system to visualize mtDNA in live cells^23^. Briefly, we introduced inducible Whi5 into haploid and diploid strains in which LacO arrays had been stably integrated into the mitochondrial genome (Fig. 2a). A constitutively expressed LacI tagged with 2×mNeon and a mitochondrial targeting sequence then bound to the LacO arrays, resulting in fluorescent foci that could be detected using confocal microscopy (Fig. 2b and Extended Data Fig. 2a). In addition, we used the fluorescent protein mKate2 targeted to the mitochondrial matrix to visualize the mitochondrial network. We then induced Whi5 expression in cells grown either on SCGE or SCD with different concentrations of β-estradiol and imaged the cells using three-dimensional (3D) confocal microscopy. Using a custom image-analysis pipeline, we segmented cells in two dimensions (2D) on the basis of bright-field images^30^, and segmented the mitochondrial network and identified mtDNA foci in 3D from fluorescence signals. Consistent with a previous report^19^, the volume of the mitochondrial network increases in proportion with cell volume, with cells grown on SCD having a smaller mitochondrial network than those grown on SCGE (Fig. 2c). We also found that the number of mtDNA foci (which we interpret as nucleoids) increases with cell volume (Fig. 2d) and mitochondrial network volume (Fig. 2e), indicating an increase in the number of nucleoids rather than an increase in mtDNA copy number per nucleoid (Extended Data Fig. 2b). In line with the reduced number of mtDNA copies per cell (Fig. 1b,c), we detected fewer nucleoids at a given cell volume when cells were grown on SCD (Fig. 2d).

### Mitochondrial diameter is independent of cell volume

**Extended Data Fig. 3:**
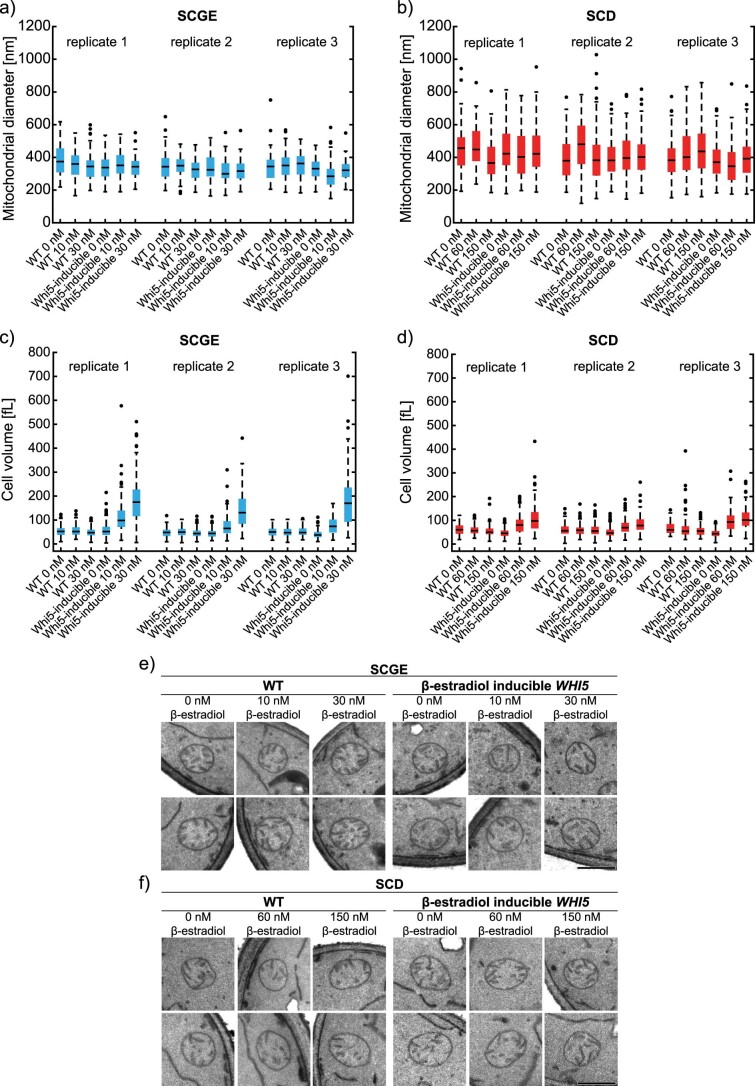
Mitochondrial diameter does not depend on cell volume in yeast. Wild-type (WT) and Whi5-inducible cells were grown in SCGE or SCD medium containing the indicated concentrations of β-estradiol, chemically fixed and analyzed by transmission electron microscopy. **a-d)** Quantification of cell volume and mitochondrial diameter corresponding to Fig. 2f. Box plots depict median (black line), 25th, 75th percentile (box). Whiskers indicate extreme values still within 1.5 interquartile ranges and outliers are depicted as single black points. Shown is the data from three independent experiments. In Fig. 2f, the mean of the means of all three replicates was plotted for the data for wild-type and Whi5-inducible cells grown without β-estradiol and Whi5-inducible cells grown in the presence of 10 nM and 30 nM (SCGE) or 60 nM and 150 nM β-estradiol (SCD). **a-b)** Mitochondrial diameter of cells grown in a) SCGE or b) SCD medium. For each sample, the diameter of 100 mitochondria was measured from electron micrographs. **c-d)** DIC images were used to calculate cell volume from cell segmentations performed with Cell-ACDC for cells grown in c) SCGE or d) SCD medium. The images were taken of live cells of the same cultures that were used for chemical fixation. **e-f)** Representative electron micrographs of mitochondria of wild-type and Whi5-inducible cells grown in SCGE (e) or SCD (f) medium containing the indicated concentrations of β-estradiol. Scale bars represent 500 nm. Source data

We observe that mitochondrial network volume, mtDNA amount, and nucleoid number all increase with cell volume. Together with previous reports on yeast^19^ and mammalian cells^20,21^, this finding suggests that the amount of mitochondria increases in larger cells but that the local mitochondrial structure is rather constant. Still, cell volume could affect mitochondrial diameter, which we cannot resolve with confocal microscopy. To test this, we analyzed haploid wild-type and Whi5-inducible cells grown in SCD or SCGE in the absence or presence of β-estradiol by transmission electron microscopy and measured mitochondrial width. We observed no changes in mean mitochondrial diameter associated with increasing cell volume, irrespective of whether the cells were grown in SCD or SCGE (Fig. 2f–h and Extended Data Fig. 3). Furthermore, we observed no obvious cell-volume-dependent alterations of the structure of the mitochondrial inner membrane (Fig. 2g,h and Extended Data Fig. 3e,f).

### Mitochondrial network scales with cell volume without mtDNA

**Fig. 3:**
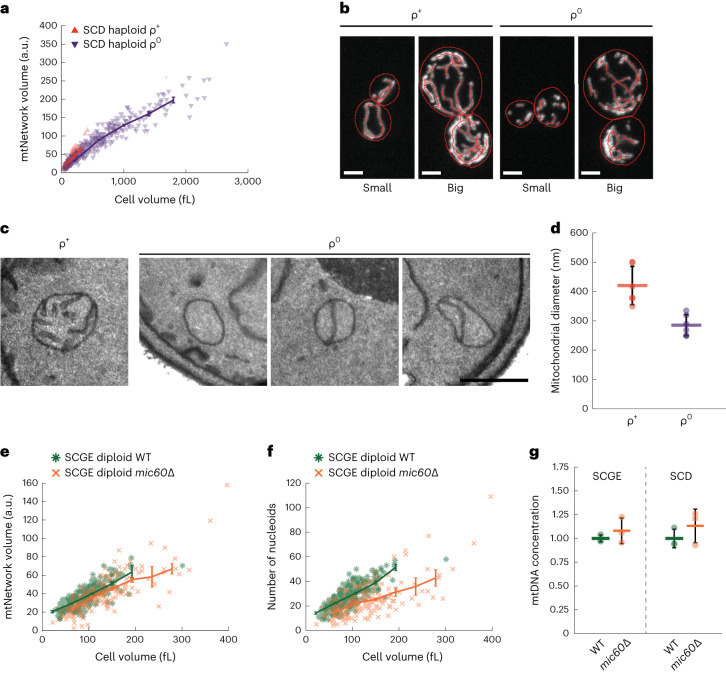
Mitochondrial network volume and mtDNA copy number are coupled to cell volume. **a**, *MIP1* was deleted in a Whi5-inducible haploid strain to generate a ρ^0^ strain. Mitochondrial network volume as a function of cell volume, compared with the parental strain (ρ^+^, data from Fig. 2c). Cells were grown on SCD. Image analysis in **a**, **e**, and **f** was performed in 3D for 3 biological replicates with *n* > 50 cells each (*n*_ρ+_ = 616; n_ρ0_ = 623 total cells). Lines connect binned means (shown at the center of the respective bin); error bars indicate s.e. (**a**,**e**,**f**). **b**, Representative confocal live-cell images (maximum intensity projections) of ρ^+^ and ρ^0^ cells without (small) or with (big) 150 nM β-estradiol are shown, with cell (red dashed lines) and mitochondrial-network segmentations. Corresponding images without network segmentation are shown in Extended Data Figure 4a. Scale bars (**b**), 2 µm. **c**,**d**, Haploid Whi5-inducible (ρ^+^) and Whi5-inducible *mip1*Δ cells (ρ^0^) were grown in SCD without β-estradiol, chemically fixed, and analyzed by transmission electron microscopy. **c**, Representative electron micrographs of mitochondria of ρ^+^ and ρ^0^ cells. Scale bar, 500 nm. **d**, Mitochondrial diameter of ρ^+^ and ρ^0^ cells (mean values of *n* = 6 biological replicates (dots) and mean of the means (line)). For each replicate, the diameters of 100 mitochondria were measured from electron micrographs. Error bars depict the s.d. of the mean. Also see Extended Data Figure 4c. **e**, Mitochondrial network volume as a function of cell volume for diploid wild-type and *mic60*∆ cells, determined from confocal images. Cells for 3 biological replicates (*n*_WT_ = 286; *n*_mic60∆_ = 182 total cells) were grown on SCGE. Absolute values of the mitochondrial-network volume measurements (a.u.) are not directly comparable with those in Figure 2. **f**, The number of nucleoids as a function of cell volume, for the same cells as in **e**. **g**, Comparison of mtDNA concentrations of wild-type and *mic60*∆ cells grown in SCGE or SCD. gDNA from the same cell populations as in e and f and Extended Data Figure 4d–h was extracted, and DNA-qPCR was performed. Cell volumes were measured with a Coulter counter. mtDNA concentration (mtDNA copy number per cell divided by cell volume) was normalized to the wild type in the respective medium. Error bars indicate the s.d. for *n* = 3 biological replicates. Source data

**Extended Data Fig. 4:**
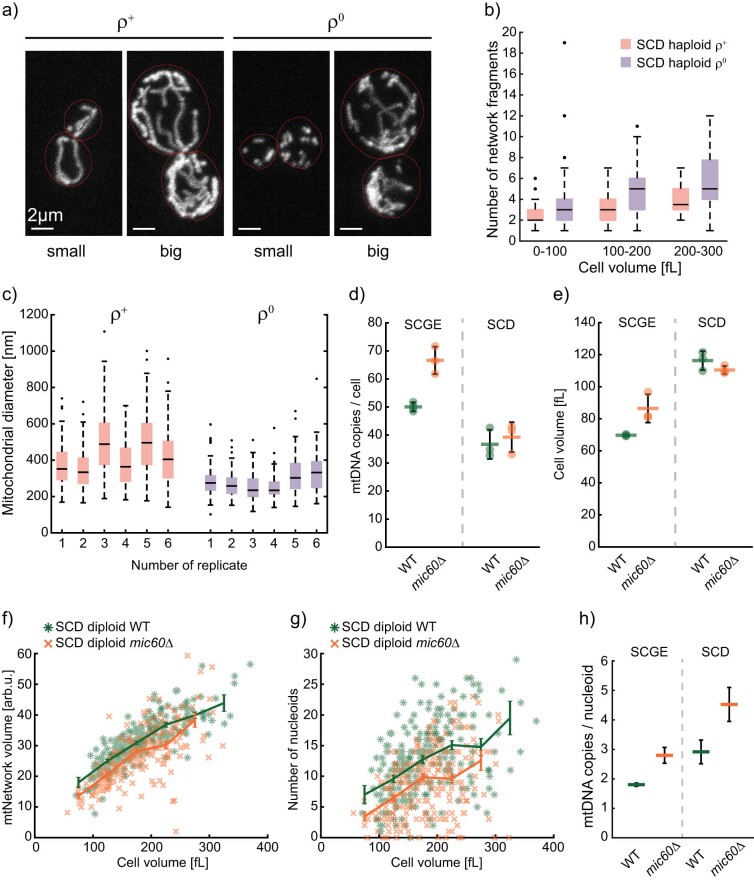
Mitochondrial network volume and mtDNA copy number are coupled to cell volume. **a)** Same images of haploid ρ^+^ and ρ^0^ cells as in Fig. 3b are shown without network segmentation. **b)** The number of separate mitochondrial network fragments in haploid ρ^+^ and ρ^0^ cells was calculated for each cell form the mitochondrial network segmentations obtained in Fig. 3a. Cell volume was binned as indicated. Box plots depict median (black line), 25th, 75th percentile (box). Whiskers indicate extreme values still within 1.5 interquartile ranges and outliers are depicted as single black points. **c)** Quantification of mitochondrial diameter for haploid ρ^+^ and ρ^0^ cells corresponding to Fig. 3d. The cells were analyzed by transmission electron microscopy and for each replicate the diameter of 100 mitochondria was measured. Shown are box plots for the single replicates. Figure 3d shows the mean of the means of all six replicates. Box plot depiction as described above. **d-e)** mtDNA copy number and cell volume for wild-type and *mic60*∆ cells corresponding to Fig. 3g. mtDNA copy number was measured by DNA-qPCR and mtDNA copy number per cell was calculated by using the budding index. Cell volume measurements were performed with a Coulter counter. Shown is the mean of n = 3 biological replicates. Error bars depict standard deviations. **f)** Mitochondrial network volume as a function of cell volume for diploid wild-type and *mic60*∆ cells, determined from confocal images as in Fig. 3e-f. Here, cells were grown on SCD. Image analysis in f-g was performed in 3D for 3 biological replicates with n > 50 cells each (n_WT_=223; n_*mic60*∆_=219 total cells). **g)** Number of nucleoids as a function of cell volume, for same cells as in f. Lines connect binned means (shown at the center of the respective bin) with error bars indicating standard errors. **h)** mtDNA copies per nucleoid for wild-type and *mic60*∆ strain, measured from n = 3 biological replicates, and obtained by dividing the average mtDNA copy number concentration from Fig. 3g by the average number of nucleoids per cell volume (measured by Coulter counter) from Fig. 3f/ Extended Data Fig. 4g. Error bars indicate propagated standard errors. Source data

So far, we have shown that larger cells have a larger mitochondrial network and an increased amount of mtDNA and nucleoids. We wondered whether the increase in mitochondrial-network volume depends on mtDNA. We therefore created a strain with deletion of the only mtDNA polymerase in yeast, *MIP1*, which results in loss of mtDNA (Supplementary Table 1). We grew this strain on SCD and measured mitochondrial network volume as a function of cell volume (Fig. 3a,b). We noticed that these cells without mtDNA, called ρ^0^ cells, show a larger variability of cell volume, in particular after Whi5 overexpression, and exhibit altered network morphology (Fig. 3a,b and Extended Data Fig. 4a,b). With transmission electron microscopy, we found that in cells with *mip1* deletion (*mip1*∆), the mitochondrial diameter was reduced (Fig. 3c,d and Extended Data Fig. 4c) and almost no normal cristae were observed. Instead, most mitochondrial cross-sections contained altered inner-membrane structures that spanned the matrix or completely lacked cristae-like inner membrane structures (Fig. 3c). This is presumably caused by the absence of mtDNA, because it has been assumed that the maintenance of normal cristae structure depends on the presence of mtDNA in budding yeast^31^.

Despite the absence of mtDNA and the altered mitochondrial morphology, the mitochondrial-network volume in *mip1*∆ cells still increases with cell volume (Fig. 3a). Moreover, at a given cell volume, the network volume of *mip1*∆ cells, as determined with confocal microscopy, was similar to that in wild-type cells (Fig. 3a). However, owing to the decreased mitochondrial diameter, which we could not reliably measure with confocal microscopy, the network volume measurements of wild-type and *mip1*∆ cells are not directly comparable. Although we thus cannot exclude that deletion of *MIP1* and the loss of mtDNA alters mitochondrial-network volume, the fact that we still observe an increase with cell volume demonstrates that the regulation with cell volume occurs either upstream or independent of mtDNA.

### mtDNA copy number is set by cell volume

To test whether mtDNA copy number is causally linked to cell or mitochondrial-network volume, we used a mutant with reduced mitochondrial-network volume. In the context of previous work^32^, we observed that *mic60*∆ cells tended to have less mitochondrial-network volume. Here, we quantitatively assessed and confirmed this phenotype (Fig. 3e–g and Extended Data Fig. 4d–g). For cells grown on either SCD or SCGE, deleting *MIC60*, a component of the MICOS complex^33–35^, leads to a lower mitochondrial network volume (as measured with confocal microscopy) at a given cell volume (Fig. 3e and Extended Data Fig. 4f) and to a smaller number of nucleoids (Fig. 3f and Extended Data Fig. 4g). However, in accordance with previous work^32^, we found that, despite the reduction of mitochondrial-network volume and nucleoids, mtDNA concentration is not reduced (Fig. 3g and Extended Data Fig. 4d,e). This suggests that the mitochondrial network and mtDNA copy number are coupled independently to cell volume. An additional layer of regulation might coordinate mitochondrial network volume and nucleoid number, such that mtDNA and nucleoid number are not strictly coupled (Extended Data Fig. 4h).

### Amount of mtDNA-maintenance factors increases with cell size

In summary, we have shown that mtDNA copy number is tightly linked to cell volume. In principle, this allows cells to maintain mtDNA concentrations during cell growth: if mtDNA copy numbers are set by cell volume at any point during the cell cycle, constant concentrations can be achieved without any dedicated regulation of mtDNA replication with cell-cycle progression. This raises the question of how mtDNA copy number is coordinated with cell volume.

The abundance of most proteins increases with cell volume, maintaining constant concentrations^36^. This is thought to be due to not just an increased abundance of ribosomes^37,38^, but also an increase of global transcription and thus amounts of mRNA^39–43^. One possible mechanism for the coupling of mtDNA number to cell volume is that, similar to most genes, nuclear-encoded mitochondrial maintenance factors might have higher expression levels in larger cells. This could then lead to larger amounts of protein, potentially including those that limit mtDNA maintenance. We therefore asked whether the expression of factors that are necessary for mtDNA maintenance increases with cell volume.

**Fig. 4:**
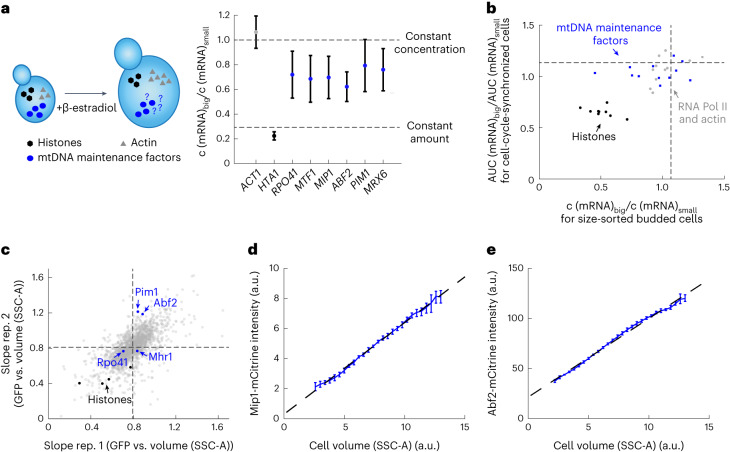
Amount of nuclear-encoded mtDNA-maintenance factors increases with cell volume. **a**, mRNA amounts of nuclear-encoded mitochondrial proteins increase with cell volume. Cells were grown on SCGE with no (small) or 30 nM (big) β-estradiol. mRNA concentrations normalized to *RDN18* (*c*(mRNA)) were measured with RT–qPCR. Shown are the ratios of concentrations in big (30 nM) and small (0 nM) haploid cell populations (means of *n* = 3 independent replicates); bars indicate propagated s.e. Cell volumes were measured with a Coulter counter to estimate the concentration ratio expected for an mRNA maintained at a constant amount. *MIP1*, *MTF1*, *RPO41*, *ABF2*, *MRX6*, and *PIM1* data were generated from RNA samples from ref. ^27^. *ACT1* and *HTA1* measurements were taken from ref. ^27^. **b**, Concentrations of nuclear transcripts encoding mtDNA-maintenance factors are largely constant regardless of changes in cell volume in two transcriptomics datasets from ref. ^36^. Ratios between big and small cells of relative expression of each transcript in size-sorted budded cells, and of the area under the curve (AUC) of relative expression during cell-cycle progression after different durations of G1 arrest, are shown for mitochondrial proteins (blue), control genes (RNA polymerase II, and *ACT1*) scaling with cell volume (gray), and histones (black). Dashed lines show medians of all transcripts. **c**, Analysis of the cell-volume dependence of GFP-fusion proteins performed in ref. ^36^, based on data by ref. ^44^. Cell-volume dependence was quantified as the normalized slope of a linear fit to the GFP intensity as a function of cell size (SSC-A) for budded cells in two biological replicates. Similar to most proteins (gray), the amount of mtDNA-maintenance factors (blue) increases with cell volume. The concentration of histones (black) decreases in big cells. Dashed lines show mean slopes for all fusion proteins included in the dataset. **d**,**e**, Flow cytometry was used to measure total cellular mCitrine fluorescence intensity in haploid strains in which either *MIP1* (**d**) or *ABF2* (**e**) was endogenously tagged. SSC-A-signal was used as a measure of cell volume (Extended Data Fig. 6). Shown are binned means after background correction using a non-fluorescent strain. Error bars indicate estimated experimental errors (see Methods). Dashed lines show linear fits to the binned means. Source data

First, we determined the dependence of transcript concentration on cell volume for several nuclear-encoded mitochondrial factors. We re-analyzed by reverse-transcription qPCR (RT–qPCR) RNA samples of a Whi5-inducible strain grown on SCGE that we have recently used to determine the concentration of histone transcripts as a function of cell volume^27^. As shown previously, the transcript concentration of the control gene *ACT1* is maintained at a nearly constant level. In accordance with the fact that the amount of mtDNA increases with cell volume, constant concentrations of *COX2* and *COX3* transcripts, two genes encoded by mtDNA, are maintained (Extended Data Fig. 1d). By contrast, the concentration of histone mRNA decreases in inverse proportion to cell volume to maintain constant amounts of histone^27^. We found that the concentration of transcripts of all analyzed nuclear-encoded mitochondrial factors only slightly decreases with cell volume, resulting in substantially increased amounts of transcripts in large cells (Fig. 4a). To validate this finding, we examined two published datasets^36^ that measured the dependence of transcripts on cell size (1) in budded cells sorted by cell size (total protein content) using flow cytometry and (2) during the first cell cycle of cells released from G1 arrests of varying lengths of time. Again, we found that, similar to most transcripts, the transcripts of factors involved in mtDNA maintenance are kept at largely cell-size-independent concentrations (Fig. 4b and Extended Data Fig. 1c).

Next, we asked whether the increasing transcript amounts lead to larger amounts of the corresponding proteins. Again, we made use of an analysis performed by Swaffer et al.^36^: using previously published flow-cytometry data on a collection of strains in which each open reading frame (where possible) was tagged with green fluorescent protein (GFP)^44^, the dependence of protein amounts on cell size was analyzed. Many mtDNA-maintenance factors were excluded from this dataset for technical reasons, in particular owing to low expression. However, for all included mtDNA factors (Abf2, Mhr1, Pim1, Rpo41), the increase in protein amount with increasing cell size was similar to or stronger than the average of that for all measured proteins (Fig. 4c).

**Extended Data Fig. 5:**
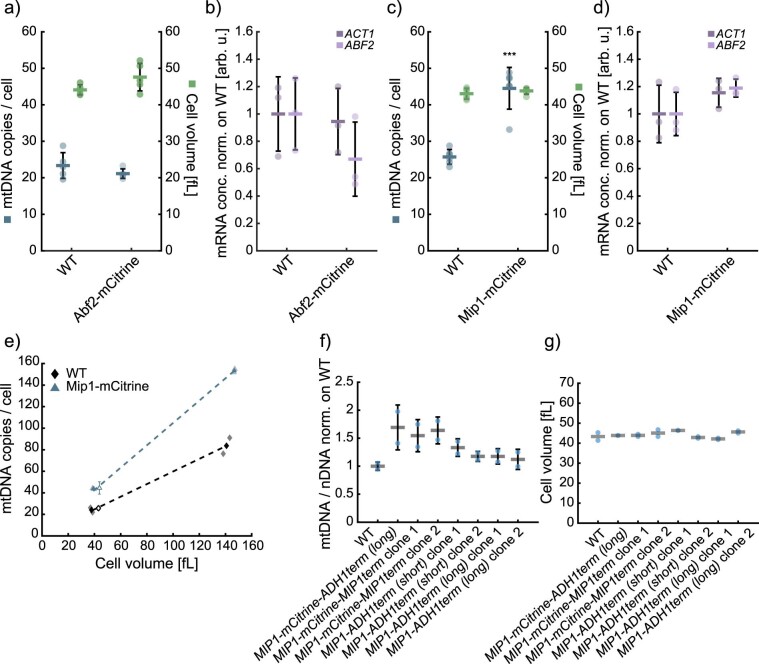
Abf2-mCitrine and Mip1-mCitrine are functional. To measure mtDNA copy numbers, mean cell volume, and transcript levels in tagged strains, DNA-qPCRs, Coulter counter measurements and RT-qPCR were performed. **a-b)** We did not observe any significant changes of mtDNA copy number, cell volume (a; n = 5) or *ACT1* and *ABF2* transcript levels (b; n = 3) upon tagging Abf2 with mCitrine. **c-d)** Tagging Mip1 with mCitrine leads to a significant (p = 0.00002; determined by a by a two-tailed t-test; * p < 0.05, ** p < 0.01,*** p < 0.001) increase of mtDNA copy number by ∼ 80% (c; n = 6), potentially due to an increased replication rate (Extended Data Fig. 8), while cell volume and transcript levels remain unchanged (d; n = 3). **e)** To test if despite the change in mtDNA copy number, the cell-volume-dependent regulation of mtDNA is still intact, we tagged Mip1-mCitrine in a Whi5-inducible strain (filled triangles; darker symbols show mean of n = 2 biological replicates (light symbols)). We find that the Mip1-mCitrine strain still shows a cell volume-dependent increase of mtDNA. Error bars indicate standard deviations (a-e). **f-g)** Increase of mtDNA copy number by mCitrine tagged Mip1 is not influenced by the terminator sequence. **f)** mtDNA copy number in Mip1 clones with varying terminator sequences normalized on wild-type. Tested was the endogenous *MIP1* with a short and a long version of the *ADH1-*terminator sequence and Mip1-mCitrine with the *MIP1* terminator sequence (Supplementary Table 5). The strain including *MIP1-mCitrine-ADH1term (long)* is the same as in c-e and Fig. 4d. **g)** Cell volume measurements for strains from f) performed with a Coulter Counter. Data for each clone consists of n = 2 biological replicates, bars show means. Source data

**Extended Data Fig. 6:**
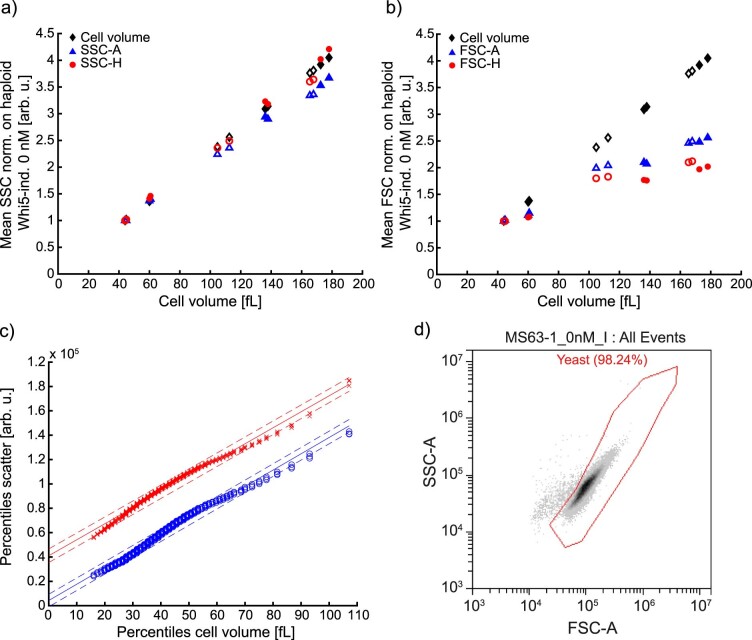
Comparison of flow cytometry side scatter and forward scatter measurements. Whi5-inducible cell populations of haploid (open symbols) and diploid cells (filled symbols) were grown on SCGE with β-estradiol (haploids 0 nM, 10 nM, 30 nM; diploids 0 nM, 25 nM, 50 nM) and measured with a CytoFlex S Flow Cytometer (Beckman Coulter) in technical duplicates, recording SSC **(a)** and FSC **(b)**. Flow cytometry measurements were compared to mean cell volumes obtained from Coulter counter measurements. Blue triangles show area and red circles height measurements, both normalized on the Whi5-inducible strain grown with 0 nM β-estradiol. Normalized Coulter counter measurements are shown to guide the eye (black diamonds). We find that both side scatter measurements correlate well with Coulter counter cell volume measurements. **c)** We anticipate that SSC-A should be proportional to cell volume independent of cell-cycle stage. To test if this is the case, we compared the percentiles of scatter signal (SSC-A: blue circles, FSC-A: red crosses) with the percentiles of Coulter counter measurements for wild-type. Data of n = 3 biological replicates are shown. Lines show linear fits to the pooled data, with dashed lines showing 95% confidence prediction intervals. **d)** Example for gating strategy. Source data

To further confirm that the amount of protein necessary for mtDNA maintenance increases with cell volume, we constructed haploid strains in which we endogenously tagged the mtDNA polymerase *MIP1* as well as the mtDNA packaging factor *ABF2* with *mCitrine*. We ensured that the tagged proteins were functional by testing growth on SCGE and measuring mtDNA concentrations with qPCR (Extended Data Fig. 5). For Mip1-mCitrine, we found increased amounts of mtDNA (Extended Data Fig. 5c). However, because the strain shows the typical increase of mtDNA copy number as cell volume increases, the mechanism ensuring dependence on cell volume is still intact, suggesting that the regulation of Mip1 with cell volume is not dramatically impaired (Extended Data Fig. 5e). Using mCitrine fluorescence intensity as a proxy for protein amount and side scatter as a measure for cell volume (Extended Data Fig. 6), we determined the cell-volume dependence of Mip1 and Abf2 protein amounts with flow cytometry. In accordance with the transcript measurements, we found that the amounts of Mip1-mCitrine and Abf2-mCitrine strongly increase with cell volume (Fig. 4d,e).

### Mip1 and Abf2 are limiting for mtDNA maintenance

**Fig. 5:**
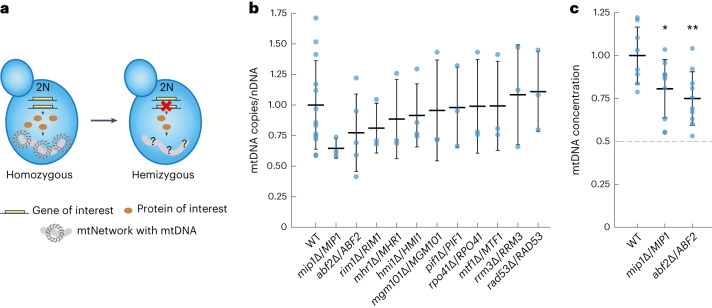
Hemizygous screen identifies factors that limit mtDNA maintenance. **a**, To reduce the expression level of potentially limiting mtDNA-maintenance factors, hemizygous diploid strains were constructed by deleting one allele of the gene of interest. **b**, Hemizygous diploid strains (genotype as indicated) were grown on SCGE. mtDNA copy numbers per nuclear DNA, as determined by DNA-qPCR, were normalized to the wild type. Lines represent the mean of at least 3 biological replicates (*n*_WT_ = 13, *n*_*abf2*∆/*ABF2*_ = 5, *n*_*hmi1*∆/*HMI1*_ = 4, *n*_other strains_ = 3 biological replicates) and error bars indicate s.d. **c**, Validation of hemizygous *MIP1* and *ABF2* strains with independent replicates of the experiment shown in **b**, shown as mtDNA concentration (mtDNA copy number per cell divided by cell volume) normalized to WT for *n*_WT_ = 8, *n*_*abf2*∆/*ABF2*_ = 10, *n*_*mip1*∆/*MIP1*_ = 10 biological replicates. Significance was determined by a two-tailed, two-sample *t*-test (*P < 0.05, **P < 0.01; *P*_*abf2*∆/*ABF2*_ = 0.0046, *P*_*mip1*∆/*MIP1*_ = 0.0271). Source data

Our results suggest that, in larger cells, the proteins required for mtDNA replication and maintenance are present at higher numbers. If those proteins limit mtDNA maintenance, meaning that a change in their abundance would cause a proportional change in the mtDNA copy number, this could explain why larger cells have more mtDNA. To test whether this is the case, and if so, which proteins are limiting, we created a series of hemizygous diploid strains. In each strain, we deleted one allele of a gene involved in mtDNA maintenance^45^, including Mip1 (ref. ^9^), Abf2 (ref. ^8^), the ssDNA-binding protein Rim1 (ref. ^46^), helicases^47–49^, and proteins involved in DNA recombination^50^. Because most budding-yeast genes do not exhibit dosage compensation at the transcript^51^ or protein level^52^, hemizygous diploids in most cases show a 50% decrease of the corresponding transcript and protein. Should this protein then be perfectly limiting for mtDNA maintenance, we would expect a 50% reduction of the mtDNA copy number (Fig. 5a).

**Extended Data Fig. 7:**
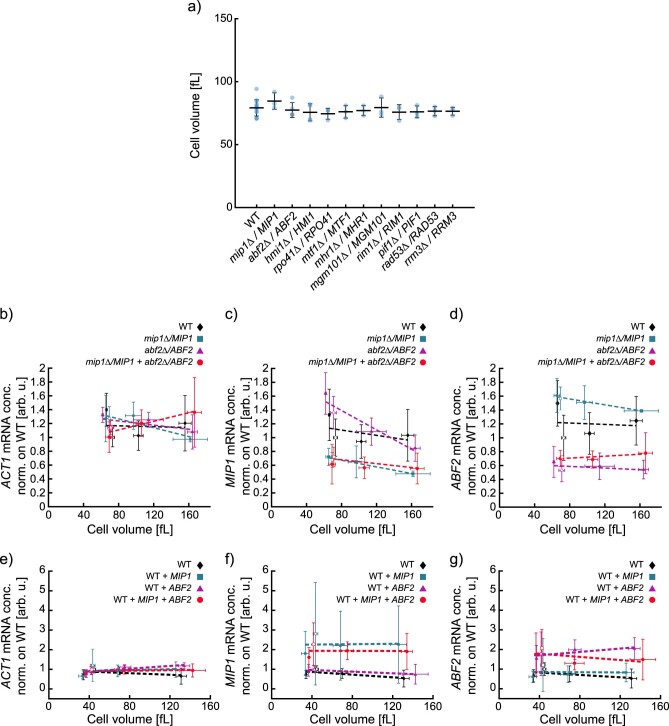
mRNA concentrations and cell volumes of hemizygous and multicopy strains. **a)** Coulter counter measurements show no significant changes in mean cell volume for hemizygous mutants from Fig. 5b compared to wild-type (n_WT_=13, n_*abf2∆/ABF2*_ = 5, n_*hmi1∆/HMI1*_ = 4, n_other strains_ = 3). **b-d)** To verify that transcript levels are not dosage compensated in hemizygous *MIP1*, *ABF2* and double hemizygous strains, mRNA concentrations were measured by RT-qPCR for Whi5-inducible (0, 15, 60 nM β-estradiol; filled symbols) and non-inducible strains (open symbols) grown on SCGE, and normalized to the non-inducible wild-type (open diamond). Shown is the mean of n_WT_ = 5 and n_Hemizygous_ = 3 biological replicates. **b)** *ACT1* concentrations stay similar for all strains. **c)** *MIP1* mRNA concentration is reduced by ∼ 50% in single and double hemizygous strains. **d)** *ABF2* mRNA concentration is reduced by ∼ 50% in single and double hemizygous strains. **e-g)** To verify that additional gene copies of *MIP1* and *ABF2* are expressed, mRNA concentrations were measured by RT-qPCR for Whi5-inducible (0, 10, 30 nM β-estradiol; filled symbols) and non-inducible strains (open symbols) grown on SCGE, and normalized on the non-inducible wild-type (open diamond). Shown is the mean of n = 3 biological replicates. **e)** *ACT1* concentrations stay comparable for all strains. **f)** *MIP1* mRNA concentrations increase by ∼ 100% in multicopy strains, where an additional *MIP1* allele was inserted. **g)** *ABF2* mRNA concentrations increase by ∼ 100% in multicopy strains, where an additional *ABF2* allele was inserted. Error bars depict standard deviations (a-g). Source data

For each hemizygous strain, we performed qPCR experiments to measure the ratio of mtDNA to nuclear DNA copies. We also measured cell volume, but did not find any major changes (Extended Data Fig. 7a). As shown in Figure 5b, we found that reducing the gene dosage of *MIP1* and *ABF2* had the strongest effect on mtDNA copy number. However, as validated by additional independent experiments (Fig. 5c), even diploids that were hemizygous for *MIP1* or *ABF2* showed a reduction to only 79% or 69%, respectively. To rule out the possibility that we do not see a reduction of mtDNA to 50% because *MIP1* and *ABF2* exhibit dosage compensation, we measured their expression level. For both hemizygotes, we did not observe clear evidence of dosage compensation (Extended Data Fig. 7b–d)—consistent with a previous study found no evidence for protein-level dosage compensation in a strain that was heterozygous for fluorescently tagged Abf2 (ref. ^52^). Taken together, the results of our work suggest that, although mtDNA copy number is sensitive to the concentration of several proteins, none of the proteins we tested is perfectly limiting.

### Mathematical model for cell-volume-dependence of mtDNA

Because we found that the cell-volume-dependent increase of mtDNA cannot be simply explained by a proportional increase of a single perfectly limiting factor, we used mathematical modeling to better understand how several partially limiting components of the mtDNA-maintenance machinery could contribute to mtDNA homeostasis. Because *MIP1* and *ABF2* hemizygotes showed the strongest reduction of mtDNA, we focused on the role of only these two proteins, neglecting the smaller contribution of other proteins.

In essence, and ignoring cell-to-cell variability, mtDNA copy number depends on the rates of mtDNA replication, degradation, and dilution by cell growth. Although Mip1 might also affect mtDNA stability through its reported exonuclease activity^53^, we aimed for a minimal model to understand the underlying principles and therefore considered only its obvious role in replication. Similarly, Abf2 might also be important for both replication and stability of mtDNA. *abf2*∆ mutants grown on fermentable medium rapidly lose mtDNA. Nevertheless, *abf2*∆ cells can be grown on non-fermentable medium, in which they can maintain a pool of mtDNA over many generations^54^, demonstrating that Abf2 is not essential for mtDNA replication. Instead, the compaction of mtDNA mediated by Abf2 seems to be important for mtDNA stability, suggesting that including this function of Abf2 in the model could be sufficient.

**Fig. 6:**
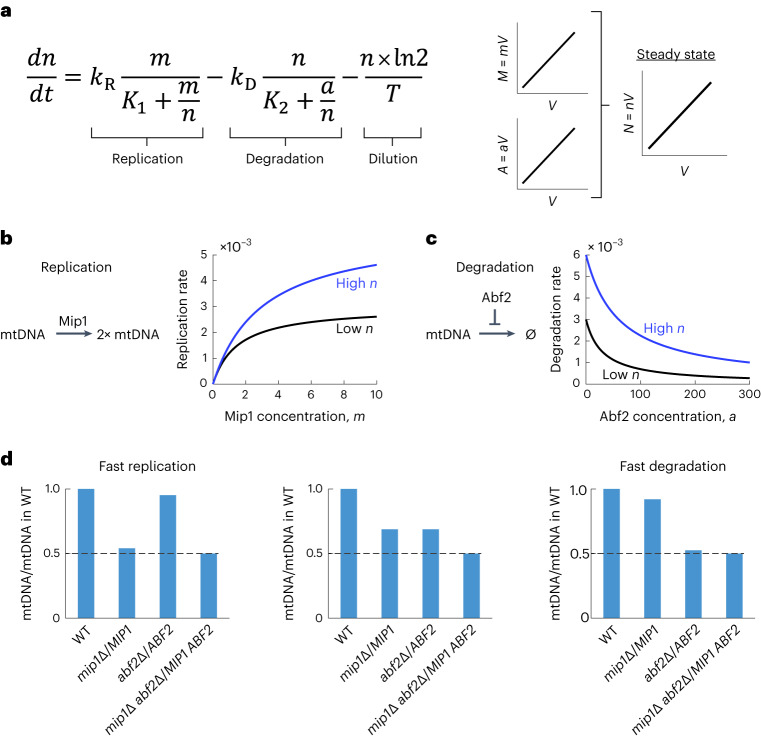
Mathematical model shows that limiting factors can couple mtDNA copy number to cell volume. **a**, The dynamic change of mtDNA concentration, *n*, is determined by mtDNA replication, degradation, and dilution due to cell growth. Cell growth is modeled as exponential with doubling time *T*. If the concentrations of Mip1, *m*, and Abf2, *a*, are constant, the corresponding protein amounts *M* and *A* increase with cell volume *V*. This results in an increase of mtDNA copy number, *N*, with cell volume. **b**, Replication is modeled as being limited by the mtDNA polymerase Mip1 at low *m*, and by *n* under saturating Mip1 concentrations. **c**, In the absence of Abf2, mtDNA degradation is modeled as exponential decay. Increasing ratios of *a* and *n* result in mtDNA stabilization, asymptotically approaching complete stability. **d**, Hemizygous deletions of *MIP1* or *ABF2* in diploid strains are modeled by reducing *m* or *a*, respectively, to 50%. Depending on the model parameters, single hemizygotes have mtDNA copy numbers between 50% and 100% of the copy numbers in the wild type. Independent of the model parameters, a double hemizygote always shows a reduction of mtDNA copy number to 50%. Source data

In the model, we account for these considerations by describing replication as a process occurring at a rate that is determined by the concentrations of mtDNA (*n*) and of the mtDNA polymerase Mip1 (*m*), such that the time derivative is $\frac{{dn}}{{dt}}={k}_{\rm R}\frac{m}{{K}_{1}+\frac{m}{n}}$ (Fig. 6a,b). Here, *k*_R_ and *K*_1_ describe the maximal rate of replication per mtDNA and the dissociation constant of Mip1 and mtDNA. We assume that, at low concentrations, most polymerase is bound to mtDNA. Thus, Mip1 alone becomes limiting, and the mtDNA replication rate is proportional to *m* and independent of *n*. At high Mip1 concentrations, mtDNA becomes saturated, and the replication rate is proportional to *n*.

Similarly, we model mtDNA degradation as a process inhibited by increasing concentrations of Abf2 (*a*), such that $\frac{{dn}}{{dt}}=-{k}_{\rm D}\frac{n}{{K}_{2}+\frac{a}{n}}$, where *k*_D_ and *K*_2_ are constants (Fig. 6a,c). We assume that Abf2 is tightly bound to mtDNA, and its stabilizing effect on mtDNA therefore depends on the Abf2/mtDNA concentration ratio. Assuming that mtDNA dilution follows an exponential-growth pattern with a doubling time *T*, we can then balance replication, degradation, and dilution to obtain $\frac{{dn}}{{dt}}={k}_{\rm R}\frac{m}{{K}_{1}+\frac{m}{n}}-{k}_{\rm D}\frac{n}{{K}_{2}+\frac{a}{n}}-\frac{n\times\mathrm{ln}\;2}{T}$ (Fig. 6a). In steady state, $\frac{{dn}}{{dt}}=0$, and we can then obtain an equation directly linking the concentration of mtDNA to that of Mip1 and Abf2.

### mtDNA-maintenance machinery can couple mtDNA to cell volume

One direct implication of this result is that, if the amounts of Mip1 and Abf2 increase in direct proportion to cell volume, thereby maintaining constant concentrations *a* and *m*, the steady-state solution for the concentration of mtDNA, *n*, is also independent of cell volume. In other words, the mtDNA copy number increases in direct proportion to cell volume (Fig. 6a). Thus, our simple model explains how an increasing amount of mtDNA-maintenance machinery in bigger cells can couple mtDNA copy number to cell volume.

Next, we asked whether our model explains the reduction of mtDNA observed in diploids hemizygous for *MIP1* or *ABF2*. In the model, deletion of one of the alleles of *MIP1* or *ABF2* can be accounted for by reducing *m* or *a*, respectively, to 50%. Solving the steady-state model, we then found that the effect of the hemizygous deletions strongly depends on the exact parameters chosen: we found parameters for which both hemizygotes cause a reduction to about 70% of mtDNA compared to the wild-type, reflecting our experimental results (Fig. 6d). However, faster replication (increased *k*_R_) will shift the system to a regime in which *MIP1* hemizygotes show an almost 50% reduction, and *ABF2* hemizygotes have nearly unchanged mtDNA amounts. By contrast, increasing the degradation rate *k*_D_ results in the opposite behavior. Thus, although our model is consistent with our experimental findings if parameters are chosen such that both the replication and degradation terms are not negligible and are sensitive to *m* and *a*, single hemizygote deletions are not well-suited to test the model. However, our model makes a prediction for the outcome of simultaneous manipulation of Mip1 and Abf2 concentrations: regardless of the chosen parameters, if *m* and *a* are changed by the same factor, the concentration of mtDNA follows proportionally (Fig. 6d, see also the ‘Model’ section in the Methods).

**Fig. 7:**
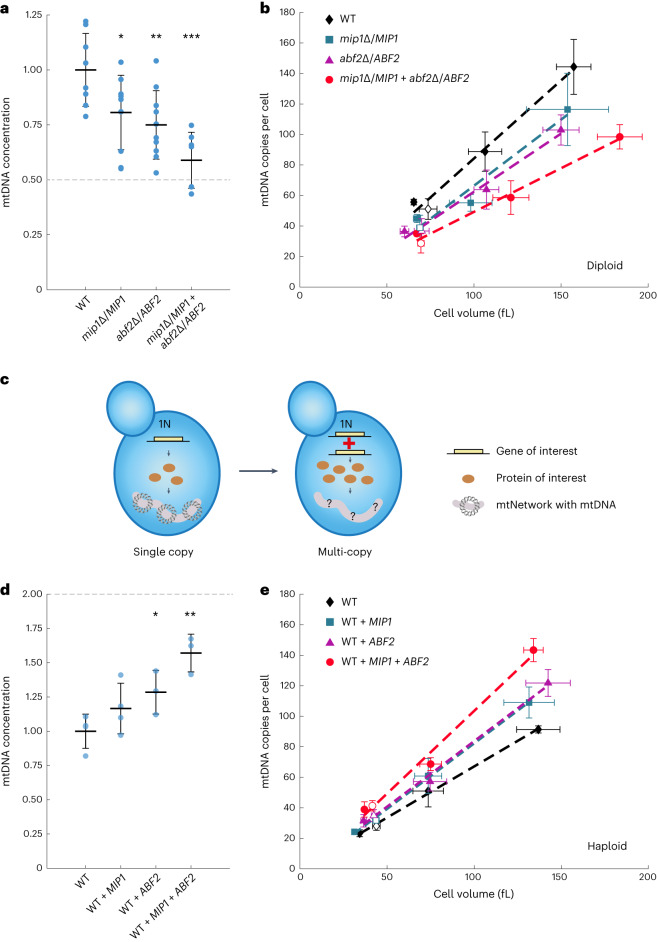
mtDNA copy number is modulated by concentrations of mtDNA-maintenance factors. **a**, mtDNA concentration, determined by DNA-qPCR of single (same data as in Fig. 5c) and double hemizygous *MIP1* and *ABF2* strains, normalized to wild type. Shown are the single replicates (blue dots) and the mean of all replicates (line). Error bars indicate the s.d. for *n*_WT_ = 8, *n*_*abf2*∆/A*BF2*_ = 10, *n*_*mip1*∆/*MIP1*_ = 10, *n*_*mip1*∆/M*IP1+abf2*∆/*ABF2*_ = 7 biological replicates. Significance was determined by a two-tailed *t*-test (*P < 0.05, **P < 0.01,***P < 0.001; *P*_*abf2*∆/*ABF2*_ = 0.0046; *P*_*mip1*∆/*MIP1*_ = 0.0271; *P*_*mip1*∆/*MIP1*+*abf2*∆/*ABF2*_ = 0.0001). **b**, mtDNA copy number per cell determined by DNA-qPCR in non-inducible (data from **a**) and Whi5-inducible hemizygous strains (*n*_WT_=4, n_*abf2*∆/*ABF2*_ = 4, n_*mip1*∆/*MIP1*_ = 3, *n*_*mip1*∆/*MIP1*+*abf2*∆/*ABF2*_ = 3 biological replicates). Error bars indicate s.d. Lines indicate linear fits of the means. **c**, Additional copies of *MIP1* and/or *ABF2* were endogenously integrated into wild-type and Whi5-inducible haploid strains. **d**,**e**, mtDNA copy number was determined by DNA-qPCR (for *n*_WT_ = 4, *n*_WT+*MIP1*_ = 4, *n*_WT+*ABF2*_ = 3, *n*_WT+*MIP1*+*ABF2*_ = 3 biological replicates), and mean cell volume was measured with a Coulter counter. **d**, mtDNA concentration normalized to the wild type for the non-inducible strains. Shown are single replicates (blue dots), the mean of all replicates (line), s.d., and significance determined by a two-tailed *t*-test (*P*_WT+*ABF2*_ = 0.0444; *P*_WT+*MIP1*+*ABF2*_ = 0.0026). **e**, mtDNA copy number per cell for Whi5-inducible and non-inducible strains. Error bars indicate s.d. Lines indicate linear fits of the means. Source data

To test this prediction, we constructed a strain that was hemizygous for both *MIP1* and *ABF2*. We verified with qPCR that expression of *MIP1* and *ABF2* was reduced to 50% (Extended Data Fig. 7b–d). As predicted, we found that the concentration of mtDNA is reduced by close to 50% (Fig. 7a). Because the model also predicts that the effect of the hemizygous deletions should be independent of cell volume, we repeated the experiments with a Whi5-inducible strain. Consistent with the model, we found that for the single and double hemizygotes, mtDNA copy numbers increase with cell volume, with similar relative reduction of mtDNA by the hemizygous deletions at all cell volumes (Fig. 7b).

Similar to a simultaneous reduction of Mip1 and Abf2 concentrations, our model predicts that the mtDNA concentration should increase by twofold if both Mip1 and Abf2 are overexpressed by twofold. To test this, we constructed haploid strains in which we endogenously integrated additional copies of the *MIP1* and/or *ABF2* genes (Fig. 7c), resulting in a twofold increase of *MIP1* and *ABF2* expression, respectively (Extended Data Fig. 7e-g). We find that overexpression of either Mip1 or Abf2 results in a moderate increase of mtDNA concentration, and simultaneous overexpression of both has an additive effect (Fig. 7d). Repeating the experiment in a Whi5-inducible strain revealed that the proportional scaling of mtDNA amount with cell volume is maintained in each strain (Fig. 7e). Importantly, simultaneous twofold overexpression of Mip1 and Abf2 results in only a 57% increase of mtDNA, which is less than the twofold increase predicted by our simple model. This suggests that, upon overexpression of the most limiting factors for mtDNA maintenance, Mip1 and Abf2, other proteins that were not included in the model become limiting. Given our analysis (Fig. 4), it seems likely that the amount of those additional factors also increases in proportion to cell volume. In contrast to the selective overexpression of only Abf2 and Mip1 we achieved through the additional gene copies, a twofold increase of cell volume would then still maintain Mip1 and Abf2 as major limiting factors, coupling mtDNA copy number to cell volume.

## Discussion

In summary, we find that in budding yeast, mtDNA copy number is tightly coupled to cell volume, both in arrested cells growing in G1 and asynchronously cycling cell populations. This is consistent with early work showing that mtDNA amount per cell increases during G1 arrest^15,18^ and with the volume of stationary cells^55^. Because the coupling of mtDNA copy number to cell volume can maintain constant mtDNA concentrations independent of cell-cycle stage, it provides an elegant mechanism for cells to maintain mtDNA homeostasis during cell growth, without requiring a coordination of mtDNA replication with the cell cycle. Interestingly, this strategy for DNA maintenance is opposite of that for nuclear DNA, whose replication is strictly coupled to cell-cycle progression but not to cell volume.

**Fig. 8:**
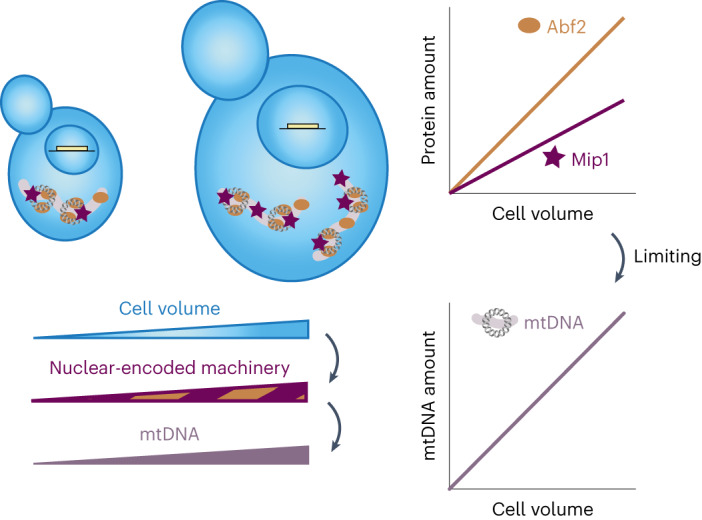
Illustration of the limiting-machinery mechanism for mtDNA homeostasis during cell growth. Cell volume sets the abundance of the nuclear-encoded mtDNA-maintenance machinery by global regulation of gene expression. This includes the mitochondrial DNA polymerase Mip1 and the packaging factor Abf2, whose amount increases with cell volume and whose abundance has a direct impact on mtDNA amounts. Thus, controlling limiting mtDNA maintenance factors by global regulation of gene expression with cell volume provides a mechanism of how mtDNA homeostasis is achieved during cell growth.

On the basis of our results, we propose a mechanism that can quantitatively explain the increase of mtDNA amount with cell volume: mtDNA concentration is determined by the rates of replication, degradation, and dilution by cell growth. Both replication and mtDNA stability can depend on mtDNA maintenance factors in a dose-dependent manner. Larger cells have overall more proteins, and also a higher amount of mtDNA replication and maintenance factors. As a consequence, larger cells can maintain more mtDNA (Fig. 8).

A single perfectly limiting mtDNA-maintenance factor could directly lead to mtDNA amounts increasing in proportion to cell volume, for example if larger cells had proportionally more mtDNA polymerase and the mtDNA replication rate increased in proportion to polymerase number. However, our experiments suggest that no single protein is perfectly limiting. Instead, we identified two partially limiting factors, Mip1 and Abf2. To obtain a conceptual understanding of how several partially limiting factors, each of which is nuclear encoded and increases in amount with cell volume, affect mtDNA numbers, we built a minimal mathematical model describing the contribution of mtDNA replication, degradation, and dilution by growth. In the model, replication and degradation are promoted and prevented, respectively, by the most limiting factors Mip1 and Abf2. We find that, even in a situation in which neither Mip1 nor Abf2 is perfectly limiting, they synergistically cause an increase in mtDNA amount proportional to cell volume. The model correctly predicts the ~50% decrease of mtDNA amount in diploids that are hemizygous for *MIP1* and *ABF2*. This suggests that, if the most limiting factors Mip1 and Abf2 are both reduced below wild-type levels, all other factors are present in excess and thus have only a weak additional limiting contribution to mtDNA maintenance. By contrast, twofold overexpression of Mip1 and Abf2 leads to a stronger deviation from the simple model, in accordance with other factors becoming partially limiting. At this point, we do not think it is helpful to extend our model to additional factors because the exact molecular mechanisms of their contribution, including rate constants, are not known. However, it seems likely that adding additional gene copies of the other limiting factors will result in a further increase of mtDNA concentration, eventually reaching a twofold increase. Because increasing cell volume likely causes an increasing abundance of all limiting factors, their combined effect is needed to fully explain the coordination of mtDNA amount and cell volume.

**Extended Data Fig. 8:**
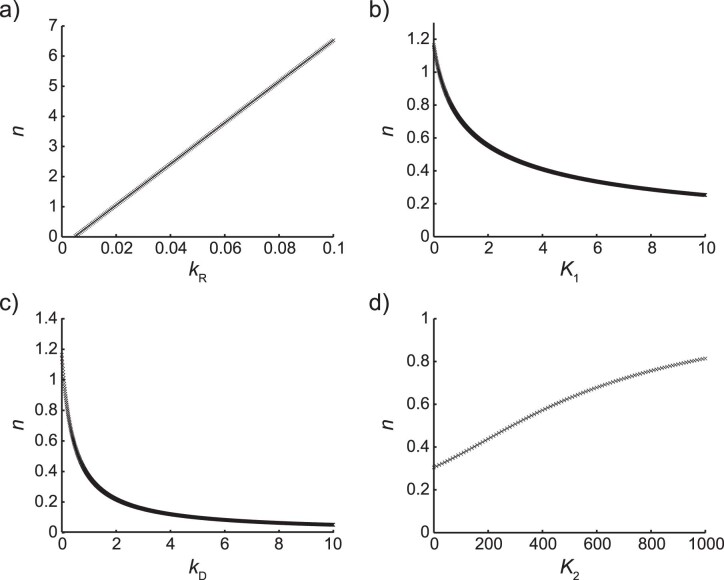
Dependence of mtDNA concentration on model rate constants. The steady state concentration of mtDNA, *n*, is modulated by all four rate constants in the model: *k*_R_ **(a)**, *K*_1_ **(b)**, *k*_D_ **(c)**, and *K*_2_ **(d)**. To illustrate the effect of each parameter, all other parameters were kept constant, respectively (*m* = 5, a = 100, *T* = 150, *k*_R_ = 0.01, *K*_1_ = 5, *k*_D_ = 1, *K*_2_ = 100).

Although we identify a mechanism coupling mtDNA amount to cell volume, additional mechanisms can modulate mtDNA homeostasis, for example as an adaptation to changing environments, such as the lower mtDNA concentration of cells grown on a fermentable medium than in those grown on a non-fermentable medium. Within the framework of the model, this can be achieved by regulating either the concentration of limiting factors or the rate constants (Extended Data Fig. 8). For example, Mrx6 was proposed to modulate mtDNA concentration through its role in degradation of factors involved in mtDNA replication^6^. Moreover, although our proposed mechanism achieves mtDNA homeostasis without cell-cycle-dependent regulation, we cannot exclude modest modulation by the cell cycle. Notably, in parasitic kinetoplastids such as *Trypanosoma* *brucei*^56^, mtDNA replication is strongly coupled to the cell cycle, suggesting that some organisms could use cell-cycle regulation as an additional level of regulation. In fact, both *MIP1* and *ABF2* expression exhibit weak cell-cycle dependence^57^, which would propagate to mtDNA amounts in our model.

Our study reveals that, in addition to mitochondrial network volume^19^, mtDNA amount increases with cell volume. We provide evidence that the coupling of the mitochondrial network volume and mtDNA to cell volume occurs through independent pathways. However, our results indicate that nucleoid number also depends directly on mitochondrial network volume. In addition, we did not observe major changes in mitochondrial diameter or ultrastructure with increasing cell volume, consistent with the weak dependence of mitochondrial structure on cell volume in mouse cells^21^. However, even in the absence of major structural changes, mitochondrial function, including respiratory activity, might be modulated by cell size. Previous work in mammalian cells has shown that, although mitochondrial mass increases with cell volume, mitochondrial function is optimal at an intermediate cell volume^21,22^. Similarly, molecular reorganization could modulate mitochondrial function in yeast such that optimal function is achieved at intermediate volumes.

In essence, the mechanism that we propose for mtDNA homeostasis requires only that the limiting components of the mtDNA-maintenance machinery increase in abundance with cell volume. One key feature is that this mechanism is robust to fluctuations in mtDNA concentration. Because steady-state concentrations of mtDNA are set by the concentrations of the nuclear-encoded limiting factors, which are themselves independent of mtDNA concentration, cells with excess or low levels of mtDNA will regress back to the steady state without an active feedback mechanism. Indeed, such passive regression to the mean has been observed for nucleoid numbers in fission yeast^24^.

We anticipate that mtDNA homeostasis achieved through limiting nuclear-encoded machinery is conserved across eukaryotes. However, the identity of the most limiting factors might vary between organisms. For example, in animals, the Abf2 homolog TFAM^7,58,59^ and the mitochondrial helicase Twinkle^10^ have a strong dose-dependent effect on mtDNA copy number. Further supporting our hypothesis, a recent study has revealed that the amount of many mitochondrial proteins, including TFAM, increases with increasing volume of human epithelial cells^60^. More generally, the increase of global protein amounts with cell size owing to increased biosynthetic capacity is widely conserved across eukaryotes. Thus, limiting nuclear-encoded genes provide a robust mechanism to achieve mtDNA homeostasis in growing cells.

## Methods

### Yeast strains

All yeast strains used in this work are derived from W303 and listed in Supplementary Table 2. Construction of yeast strains was performed with standard methods; plasmids are listed in Supplementary Table 3. Transformants were verified by control PCRs and sequencing.

Microscopy strains (haploid, diploid, ρ^0^) were generated from parental strains containing LacO arrays in mtDNA (yCO380, yCO381)^23^. Endogenous *WHI5* was deleted in yCO380 and β-estradiol-inducible *WHI5* was integrated (KSE113-1), followed by endogenous integration of a plasmid carrying the β-estradiol transcription factor (FRP880)^25^, resulting in strain ASY11-2B. Next, the plasmid ASE001-5 containing *mKate2* and *LacI* tagged with two copies of *mNeon* was integrated into the HO locus to obtain strain ASY13-1. To generate the diploid strain ASY15-1, yCO381 was transformed with ASE001-5 and crossed with ASY11-2B.

### Yeast culturing

All strains were grown at 30 °C in a shaking incubator at 250 r.p.m. (Infors, Ecotron).

Prior to growing cells on non-fermentable medium (synthetic complete medium containing 2% glycerol and 1% ethanol, SCGE), strains were grown for at least 6 h on YPD. Then, cells were washed with SCGE and transferred into SCGE. Cultures were grown for about 24 h in exponential phase when directly used for experiments, or were grown for at least 12 h before β-estradiol was added to Whi5-inducible strains. To tune Whi5 concentration in Whi5-inducible strains, cells were grown for another 24 h in the presence of the respective β-estradiol concentration. For haploid strains, concentrations of 0 nM, 10 nM, and 30 nM of β-estradiol were used; for diploids, concentrations of 0 nM, 15 nM, and 60 nM were used.

When fermentable medium was used, cells were directly inoculated in synthetic complete medium containing 2% dextrose (SCD) and were grown for at least 12 h before β-estradiol was added. For both haploid and diploid strains, concentrations of 0 nM, 15 nM, 60 nM, and 150 nM β-estradiol were added, and cells were grown for an additional 24 h.

For the G1 arrest (Fig. 1c), a *cln1/2/3* deletion strain, in which Cln1 was expressed using a β-estradiol-inducible promoter, was used^29^. Before G1 arrest, cells were grown at least 6 h on YPD with 30 nM β-estradiol, transferred into SCGE with 30 nM β-estradiol, and grown for about 24 h. To perform experiments in fermentable conditions, cells were directly inoculated in SCD medium with 60 nM β-estradiol and grown for 24 h. To initiate G1 arrest, cells were washed with the respective medium without hormone, and cultures were then collected every hour (SCGE, 0–8 h; SCD, 0–6 h).

Steady-state exponential-growth conditions were obtained by regularly measuring optical densities using a spectrophotometer (NanoDrop One^C^, Thermo Fisher Scientific) and ensuring that the optical density at 600 nm (OD_600_) was <1 through appropriate dilutions. To determine mean cell volumes of cell populations, cell-volume distributions were measured using a Coulter counter (Beckman Coulter, Z2 Particle Counter) after sonication. Samples were measured twice with two different settings (Range 1: 10–328 fL, gain: 256, current: 0.707 ma; Range 2: 328–1,856 fL, gain: 256, current: 0.125 ma). We then used both measurements to calculate a mean volume within the combined cell volume range.

### mtDNA copy number measurements

Cells were cultivated in 50 mL of the respective medium with corresponding β-estradiol concentrations. Prior to collection, cell volume distributions and optical density were measured. Cell cultures were spun at 3,400*g*, and pellets were washed with 1 mL double-distilled water.

gDNA was extracted by phenol-chloroform-isoamyl alcohol (PCI) extraction. More precisely, cells were mechanically disrupted by vortexing at 3,000 oscillations per minute (Mini-BeadBeater 24, 230 V, BioSpec Products) with glass beads in 200 µL DNA extraction buffer, pH 8.0 (2% Triton X-100, 1% SDS, 100 mM NaCl, 10 mM TRIS, 1 mM EDTA) and 200 µL PCI. After centrifugation at 16,000*g*, the aqueous phase was taken and gDNA was precipitated with 500 µL 100% ethanol. Centrifugation was then repeated, and the pellet was washed with 800 µL 70% ethanol.

To remove RNA residues, the pellet was dissolved in nuclease-free water, treated with 1 mg mL^–1^ RNase A (DNase-free), and incubated for 30 min at 37 °C. Subsequently, DNA extraction buffer and PCI were added, and extraction steps were repeated. DNA concentrations were determined with a spectrophotometer (NanoDrop One^C^, Thermo Fisher Scientific) through measurements at 260 nm. For qPCR, 1 ng DNA was used.

qPCR was performed on a LightCycler 480 Multiwell Plate 96 (Roche). For amplification, a DNA-binding fluorescent dye (BioRad, SsoAdvanced Universal SYBR Green Supermix) and specific primers for the nuclear DNA (nDNA) genes *ACT1*, *MIP1*, and *MRX6* and the mtDNA genes *COX2* and *COX3* (Supplementary Table 4) were used. For strains in which *MIP1* copy number was manipulated, *MIP1* primers were omitted from the analysis. The initial denaturation time was set to 10 min. Each sample was measured in technical triplicates. For further analysis, mean C_q_ values of the technical replicates were used. Single technical replicates were excluded from the analysis when the s.d. was higher than 0.5.

**Extended Data Fig. 9:**
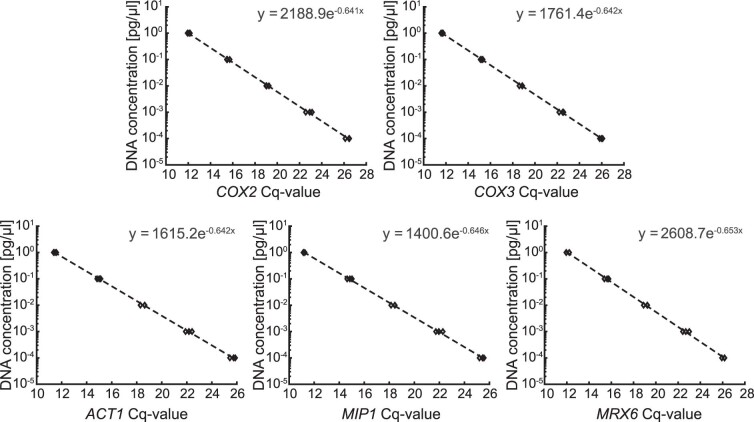
Standard curves of calibration standard. A calibration standard was constructed by fusing the amplified sequences of *COX2*, *COX3*, *ACT1*, *MIP1* and *MRX6* to one template with PCR. Then a dilution series was prepared and qPCR was performed in three replicates for each primer pair. Results are shown in a semilog plot. By fitting the data, the equation of the standard curve was obtained and used to calculate concentrations of the corresponding genes for all DNA qPCR data. Source data

To correct for differences in primer efficiencies and enable absolute measurements of DNA concentrations, a calibration standard was obtained by constructing a single PCR product containing all amplified sequences. A standard dilution series with defined input concentrations (1 × 10^–4^–1 pg µL^–1^) was then performed to obtain a standard curve for each primer pair. A linear fit to these calibration curves was finally used to calculate concentrations from qPCR measurements (Extended Data Fig. 9).

Concentrations of each gene were calculated, and nDNA concentrations (based on *ACT1*, *MIP1*, *MRX6*) and mtDNA concentrations (*COX2*, *COX3*) were pooled by calculating the means. mtDNA concentrations were then normalized to nDNA, to obtain the relative mtDNA copy number per nDNA. By counting buds through visual inspection, a budding index (the percentage of budded cells, %buds) was determined for each cell population and used to calculate the average nDNA amount per cell: $\frac{\rm{nDNA}\left(\rm{haploids}\right)}{\rm{cell}}=\frac{\left( \% \rm{buds}\times 2\right)+\left( \% \rm{no}-\rm{buds}\times 1\right)}{100}$ or $\frac{\rm{nDNA}\left(\rm{diploids}\right)}{\rm{cell}}$ $=\frac{\left( \% \rm{buds}\times 4\right)+\left( \% \rm{no}-\rm{buds}\times 2\right)}{100}$. Here, %no-buds refers to the percentage of unbudded cells. Multiplication of this average nDNA amount per cell with mtDNA copies per nDNA then allowed us to determine the average mtDNA copy number per cell.

For statistical analyses, we performed a Shapiro–Wilk test at a confidence level of *α* = 0.05 to test whether the distributions were normally distributed.

### mRNA measurements

RNA samples in Figure 4a were taken from experiments performed by Claude et al.^27^ (Fig. 2d). Briefly, cells were cultivated in 25 mL of the respective medium (YPD and then SCGE) and grown as described above. RNA was extracted by hot acidic phenol (Sigma-Aldrich) and chloroform (Thermo Fisher Scientific) extraction. RNA extraction in Extended Data Figures 1, 5, and 7 was performed with the YeaStar RNA Kit (Zymo Research), following the instructions of the given protocol. DNA contaminations were removed by a DNA digestion step using DNaseI (Life Technologies). cDNA was synthesized using 1,000 ng total RNA and random primers, following the high-capacity cDNA reverse-transcription kit protocol (Thermo Fisher Scientific). mRNA expression levels of *ACT1* and *HTA1* were taken from Claude et al.^27^ (Fig. 2d). mRNA expression levels of *MIP1*, *ABF2*, *PIM1*, *MTF1*, *RPO41*, and *MRX6* were measured by qPCR using the fluorescent dye SybrGreen for detection. Two microliters of a 1:10 dilution of cDNA was used, except for the ribosomal RNA *RDN18*, for which 2 µL of a 1:200 dilution was used. Each sample was measured in triplicate, and concentrations were calculated after normalization to *RDN18*.

### Analysis of transcript and protein cell-size dependence based on Swaffer et al.^36^

To compare the cell-size dependence of mtDNA-maintenance-factor transcripts (*ABF2*, *HMI1*, *MGM101*, *MHR1*, *MIP1*, *MRX6*, *MTF1*, *PIF1*, *PIM1*, *RAD53*, *RIM1*, *RPO41*, *RRM3*) with that of scaling control genes (*ACT1* and the RNA polymerase II subunits *RPB2*, *RPB3*, *RPB4*, *RPB5*, *RPB7*, *RPB8*, *RPB9*, *RPB10*, *RPB11*, *RPO21*) and the sub-scaling histones (*HHF1*, *HHF2*, *HHO1*, *HHT1*, *HTA1*, *HTA2*, *HTB1*, *HTB2*, *HTZ1*), we analyzed two datasets published by Swaffer et al.^36^. For the first dataset, budded cells were sorted into four size bins using a total protein stain as a measure for cell size and were then analyzed with RNA sequencing. We compared the ratio (mean of two independent replicates) between the relative expression levels in the largest and smallest cells. For the second dataset, cells were elutriated and arrested in G1 for different amounts of time before synchronous release, resulting in different cell volumes at the time of cell-cycle entry. The temporal evolution of the transcriptome during cell-cycle progression was then analyzed with RNA sequencing. The relative expression throughout the cell cycle was calculated as the AUC of the expression-level time course (after applying a spline). We compared the ratio between the largest and smallest cells (mean of two independent replicates). On the basis of combined analysis of both datasets, all mtDNA factors that we analyzed were classified as ‘scaling’ by Swaffer et al. In the first dataset (but not the second), *RAD53* showed a strongly increased expression in big cells, which we attributed to its strong cell-cycle dependence. We therefore excluded *RAD53* from further analysis.

Of the mtDNA-maintenance factors described above, Abf2, Mhr1, Pim1, and Mhr1 are included in the Swaffer et al. analysis of the dependence of protein amount on cell volume, on the basis of flow-cytometry measurements of strains carrying GFP-tagged alleles of the respective proteins performed by Parts et al.^44^. Briefly, the normalized slope of GFP intensity as a function side scatter was used to estimate the cell-volume-dependence of the protein amount. Proteins maintained at a perfectly constant amount would be expected to exhibit a slope of 0, whereas proteins maintained at a constant concentration would exhibit a slope of 1. Parts et al. performed two independent biological replicates, which we analyzed separately.

### Microscopy

For imaging, coverslips (µ-Slide 8 Well, ibi-Treat, ibidi) were covered with 200 µL concanavalin A (conA, 1 mg mL^–1^ in H_2_O) and incubated for 5–10 min. The wells were then washed twice with water and air dried.

Cells were cultivated as described in 5 mL medium. Then, 1 mL of the culture was sonicated and 200 µL was transferred to the conA-covered well. The cells were then allowed to settle for about 5 min before the supernatant was removed and the wells were washed twice with medium. Then 200 µL medium was used to cover the wells.

Live-cell fluorescence microscopy experiments were performed on a Zeiss LSM 800 confocal microscope (software: Zen 2.3, blue edition) equipped with an Axiocam 506 camera, using the confocal mode. Images were taken using a 63x/1.4 Oil DIC objective. *Z*-stacks were acquired over 15.05 µm in 0.35-µm increments. mKate2 was imaged with an excitation wavelength of 561 nm, and detecting emission between 610 and 700 nm. mNeon was excited at 488 nm and detected between 410 and 546 nm. Bright-field images were taken using the transmitted light detector (T-PMT).

### Cell segmentation

Cell segmentation was performed using Cellpose v0.6 (ref. ^61^), with the ‘cell diameter’ parameter set to 50 pixels, ‘flow threshold’ set to 0.4, and ‘cell probability threshold’ set to 0. Cell-ACDC^30^ was used to manually correct segmentation, annotate buds to their corresponding mother cells, and calculate cell volume.

### Nucleoid counting and mitochondrial network segmentation

To count the number of nucleoids and compute the mitochondrial network volume from confocal 3D Z-stack images, we developed a custom routine written in Python (https://github.com/SchmollerLab/SeelMito/releases/tag/v1.0). Mitochondrial network segmentation and spot detection were performed in 3D.

The analysis steps are: (1) Application of a 3D gaussian filter with a small sigma (0.75 voxel) of both the nucleoids and mitochondria signals. (2) Instance segmentation of the mitochondria signal using automatic Li thresholding^62^ (threshold_li function from the library scikit-image^63^). (3) Normalization of the mitochondria signal using the median of the voxel intensities classified as mitochondria in step 2. (4) 3D local maxima detection (peaks) in the nucleoids signal using the peak_local_max function from the Python library scikit-image. (5) Discarding of peaks that are below a threshold value determined with the automatic Li thresholding algorithm. (6) Discarding of overlapping peaks. If two or more peaks are within a resolution-limited volume, only the peak with highest intensity was retained. The resolution-limited volume was determined as a spheroid with $x$ and $y$ radii equal to the Abbe diffraction limit and the $z$ radius equal to 1 µm. With a numerical aperture of 1.4 and mNeon emission wavelength of about 509 nm, the resolution-limited volume has radius $x=y=0.222\,{\rm{\mu m}}$. (7) The remaining peaks undergo a subsequent iterative filtering routine. (a) Each voxel classified as mitochondria in step 2 is further classified as inside or outside of the nucleoids. A voxel is outside of the nucleoid if it is not within the resolution-limited volume centered at the peak coordinates. (b) The nucleoids signal is normalized by the mean of the voxel intensities classified as outside of the nucleoids (step 7a). (c) The normalized intensity distribution of the voxels inside each nucleoid volume is compared with the same voxels from the mitochondria signal. The comparison is performed with a Welch’s *t*-test and if the *P* value is above 0.025 or the *t*-statistic is negative (that is, the mitochondria signal is higher than the nucleoid signal), the peak is discarded. Here, we verified that the analysis is robust to choosing different *P* values (0.001 or 0.05). (d) Steps a to c are repeated until the number of nucleoids stops changing. The assumption of comparing the nucleoids signal to the mitochondria signal is that a peak is a valid nucleoid only if it has an intensity that is significantly higher than the corresponding mitochondria signal (after normalization).

The resulting peaks are considered valid nucleoids and are therefore counted. The mitochondrial network volume is computed as the sum of the voxels classified as mitochondria in step 2. Note that owing to the optical resolution limit, the width of the network is not measured accurately with confocal microscopy and the obtained mitochondria network volume is therefore not an absolute measure for the physical volume of the mitochondria. To enhance visualization of the mitochondrial network in the representative microscopy images in Figures 2b and 3b, we computed the skeletons from the 3D network semantic segmentation masks. This was achieved with the Lee algorithm^64^ (implemented in the Python library scikit-image). Briefly, this algorithm performs several morphological operations (erosions) aimed at thinning the segmentation volume to a single-pixel wide skeleton. Skeletons were used only for visualization purposes.

**Extended Data Fig. 10:**
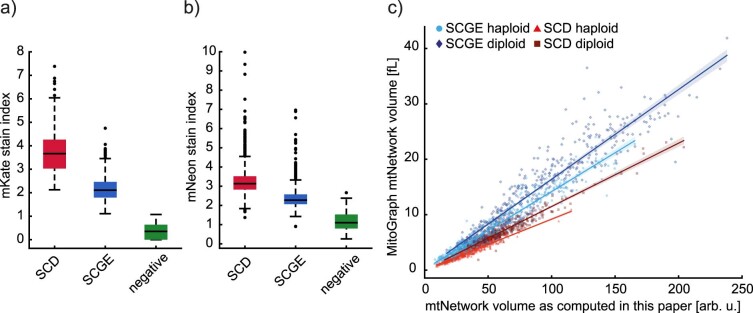
Validation of mitochondria and mtDNA image analysis. Comparison of the mKate **a)** and mNeon **b)** stain indexes between positive control (pooled data from Fig. 2b-e grouped into cells grown in SCD and SCGE medium) and negative control (haploid strain grown in SCGE). The negative control lacks both mKate and mNeon fluorescent proteins. The stain index is computed as the median of the intensities from the positive pixels minus the median of the intensities from the negative pixels, all divided by the standard deviation of the intensities from the negative pixels. The positive pixels are the pixels inside the segmented structures, where mitochondria network was labeled with mKate and nucleoids with mNeon. The negative pixels are the pixels inside each segmented cell but outside of the segmented structure. Box plots depict median (black line), 25th, 75th percentile (box). Whiskers indicate extreme values still within 1.5 interquartile ranges and outliers are depicted as single black points. **c)** Correlation plot between the mitochondria network computed in this paper (see “Nucleoid counting and mitochondrial network segmentation” in the methods section) and the mitochondria network computed by the software MitoGraph in both SCD and SCGE medium for haploid and diploid WT cells. Thick lines are linear regressions and the shaded area indicates 95% confidence intervals computed with 1000 bootstrap resamples. Pearson’s correlation coefficients are 0.95, 0.96, 0.94, 0.96, for SCD diploid, SCD haploid, SCGE diploid, and SCGE haploid respectively, indicating a very strong correlation between the two methods. Source data

To verify that the mitochondrial network and nucleoid detection algorithm detects real signal, we computed the stain index, and compared it with a negative control without fluorescent proteins (Extended Data Fig. 10a,b). Through comparison with our qPCR results, and supporting the accuracy of our nucleoid detection algorithm, we find that, on average, each nucleoid contains about two copies of mtDNA, which is in agreement with previous studies^6,65^ (Extended Data Fig. 2b). Moreover, we compared the results of our mitochondrial network quantification with that obtained with MitoGraph^19,66^, and found very high correlation (Extended Data Fig. 10c).

Finally, to compute the number of mitochondrial network fragments shown in Extended Data Figure 4b, we labeled the 3D network semantic segmentation masks using connected-component labeling to obtain the corresponding instance segmentation masks. This allows counting the number of distinct objects (fragments) within each cell.

### Electron microscopy

For cell-volume quantification, a sample was taken from each culture before the cells were prepared for electron microscopy. These samples were analyzed by light microscopy, and DIC images of living cells were taken using a Zeiss Axiophot microscope equipped with a Plan-Neofluar 100x/1.30 Oil objective (Carl Zeiss Lichtmikroskopie) and a Leica DFC360 FX camera operated with the Leica LAS AF software version 2.2.1 (Leica Microsystems). Cell segmentation and volume estimation were performed using Cell-ACDC^30^ as described above.

Fixation of yeast cells for electron microscopy with glutaraldehyde and potassium permanganate was performed as described in ref. ^67^ with the following changes: glutaraldehyde fixation was performed with 3% glutaraldehyde, 0.1 M sodium cacodylate, 1 mM CaCl_2_, pH 7.2, and the samples were subsequently washed with 0.1 M sodium cacodylate, 1 mM CaCl_2_, pH 7.2. Treatment with potassium permanganate was either performed before (cells grown in SCD) or after (cells grown in SCGE) embedding in agar. After treatment with sodium metaperiodate and overnight staining with 2% uranyl acetate at room temperature, dehydration of chemically fixed yeast cells with ethanol and propylene oxide, Epon infiltration, and contrast enhancement of ultrathin sections were essentially performed as described in ref. ^68^, with the following modifications: all dehydration steps were performed at 4 °C, Epon infiltration was performed at room temperature, and contrast enhancement of ultrathin sections was performed for 15 min with 2% uranyl acetate and for 3 min with lead citrate. Electron micrographs were taken using a JEOL JEM-1400 Plus transmission electron microscope operated at 80 kV, a 3,296 × 2,472 pixels JEOL Ruby CCD camera, and the TEM Center software, either v1.7.12.1984 or v1.7.19.2439 (JEOL). As an estimate of mitochondrial diameter, the length of the minor axis of 100 mitochondria was measured for each sample from electron micrographs using Fiji^69^.

### Flow cytometry

Wild-type cells in which Mip1 or Abf2 were endogenously tagged with mCitrine were analyzed with flow cytometry to determine the dependence of Mip1 and Abf2 protein amounts on cell volume.

Cells were cultured as described above. After 16–20 h of growth on SCGE, cultures were diluted and split into three technical replicates. For control measurements shown in Extended Data Figure 6a,b, ß-estradiol was added. Optical density was measured with a spectrophotometer (NanoDrop One^C^, Thermo Fisher Scientific) and only cultures with OD_600_ < 0.9 were included in the flow cytometry measurements. Cultures were kept on ice until measurement. After sonication for 10 s, the mean cell volume of each culture was determined using a Coulter counter. The flow cytometry measurement was performed on a CytoFlex S Flow Cytometer (Beckman Coulter) with CytExpert 2.4 and the parameters FSC-A, SSC-A, and total fluorescence intensity using the FITC channel (excitation at 488 nm and detection with a 525/40 nm filter) were recorded. Cells were analyzed at a slow flow rate (10 µL min^–1^) and data were collected from 50,000 events per sample. Through a standard gating strategy (Extended Data Fig. 6d), cell debris, particles, and doublets were excluded from the analysis. Identical settings were used for all measurements. To correct for the autofluorescence of yeast cells, the parent strain without the mCitrine tag was measured.

After confirming that differences between technical replicates were negligible, the three replicates measured on one day were pooled and binned according to SSC-A, which is a good proxy for cell volume (Extended Data Fig. 6). To correct for autofluorescence, for each bin, the mean signal of the autofluorescence control was subtracted from the mean signal of the fluorescent strain in the same bin. This analysis was repeated for data obtained on a different day (three technical replicates each). Background-corrected signals obtained on each day were then averaged. For each bin, the maximum (minimum) of the two signals plus (minus) the s.e. associated with the measurement of the fluorescent strain was used to obtain an estimate of the experimental error.

### Model

To better understand the effect of limiting mtDNA-maintenance machinery on cell-volume-dependent mtDNA concentration (*n*), we built a minimal mathematical model, neglecting cell-to-cell variability and any potential contributions of asymmetric mtDNA inheritance between mother cells and their buds. We assumed that the rate of mtDNA replication is given by the concentrations of mtDNA polymerase Mip1 (*m*) as well as the mtDNA concentration, such that the synthesis rate can be described by $\frac{{dn}}{{dt}}={k}_{\rm R}\frac{m}{{K}_{1}+\frac{m}{n}}$. In the limit of saturating Mip1 concentrations, the synthesis rate approaches the constant *k*_R_ multiplied by the concentration of mtDNA. At low Mip1 concentrations, under which we assume that most Mip1 is bound to mtDNA, replication is limited by the polymerase Mip1 and thus the synthesis rate increases in direct proportion with *m*. *K*_1_ describes the dissociation constant of Mip1 and mtDNA. In addition, we assume that in the absence of Abf2, each mtDNA molecule is degraded with a rate $\frac{{k}_{\rm D}}{{K}_{2}}$, where *k*_D_ and *K*_2_ are again constants. Increasing concentrations of Abf2 (*a*) then stoichiometrically protect mtDNA from degradation, such that the total rate of mtDNA degradation can be described by $\frac{{dn}}{{dt}}=-{k}_{\rm D}\frac{n}{{K}_{2}+\frac{a}{n}}$. Here, we assume that most Abf2 is bound to mtDNA, and that its stabilizing effect depends on it being bound. As a consequence, the degradation rate depends on the stoichiometry between Abf2 and mtDNA.

Finally, we account for the fact that mtDNA is diluted by cell growth by assuming exponential growth with a doubling time *T*. Combining the contributions of replication, degradation, and dilution, we then find that:

$$\frac{dn}{dt}={k}_{\rm R}\frac{m}{{K}_{1}+\frac{m}{n}}-{k}_{\rm D}\frac{n}{{K}_{2}+\frac{a}{n}}-\frac{n\times \ln 2}{T}$$

In steady state, $\frac{{dn}}{{dt}}=0$, so that:

$$0={k}_{\rm R}\frac{m}{{K}_{1}+\frac{m}{n}}-{k}_{\rm D}\frac{n}{{K}_{2}+\frac{a}{n}}-\frac{n\times \ln 2}{T}$$

From this equation, it can be immediately seen that as long as the concentrations of Mip1 and Abf2 are constant, that is that the amounts of Mip1 and Abf2 increase in proportion to cell volume, then the concentration of mtDNA is maintained constant, that is the mtDNA copy number increases in proportion to cell volume.

In addition to the trivial solution $n=0$, the steady-state equation leads to a second-order polynomial equation:

$$\begin{array}{l}{n}^{2}({K}_{1}{K}_{2}\,\ln 2+{K}_{\rm D}{K}_{1}T)+n(a{K}_{1}\,\ln 2-{K}_{2}m{K}_{\rm R}T+{K}_{2}m\,\ln 2+{K}_{\rm D}mT)\\+(am\,\ln 2-am{K}_{\rm R}T)=0\end{array}$$

To understand the impact of hemizygous *MIP1* and *ABF2* deletions, we then chose specific parameters (‘wild-type’: *m* = 5, *a* = 100, *T* = 150, *k*_R_ = 0.01 or 0.1, *k*_D_ = 1 or 10, *K*_1_ = 5, *K*_2_ = 100), and solved the steady-state equation using *Matlab*. Although the model is not meant to accurately reflect the quantitative details of budding yeast cells, the parameters are chosen such that the relative ratios of *m*, *a*, and *n* are roughly in the range expected from our measurements and previous estimates^70^.

If *m* and *a* are increased or decreased by the same factor, as for example in the double hemizygous strain, our model leads to a proportional change of the mtDNA concentration *n*. This would be true for any other model of the form:

$$\frac{dn}{dt}=n\,f\left(\frac{m}{n}\right)-n\,g\left(\frac{a}{n}\right)-\frac{n\times \ln 2}{T}$$

in which the replication and degradation terms $f\left(\frac{m}{n}\right)$ and $g\left(\frac{a}{n}\right)$ depend only on the ratios of Mip1 and Abf2 concentrations, respectively, and mtDNA concentration. Deviations from such behavior would for example occur, if our assumption that at low Mip1 concentrations unbound Mip1 can be neglected, does not hold true.

### Reporting summary

Further information on research design is available in the Nature Portfolio Reporting Summary linked to this article.

## Online content

Any methods, additional references, Nature Portfolio reporting summaries, source data, extended data, supplementary information, acknowledgements, peer review information; details of author contributions and competing interests; and statements of data and code availability are available at 10.1038/s41594-023-01091-8.

### Supplementary information

Supplementary Information Supplementary Tables 1–5

Reporting Summary

### Source data

Source Data Figs. 1–5 and 7 and Extended Data Figs. 1–7, 9, and 10 Source data file for all main and Extended Data figures

## Data Availability

Source data for Figures. 1–5 and 7 and Extended Data Figures 1–7, 9, and 10 are provided with this paper. Raw fluorescence-microscopy data are available at: https://www.ebi.ac.uk/biostudies/BioImages/studies/S-BIAD709. Strains and other data that support this study are available from the corresponding author upon reasonable request. Source data are provided with this paper.

## Code availability

Cell-ACDC is available at: https://github.com/SchmollerLab/Cell_ACDC. Custom code used for quantification of mitochondrial networks and nucleoids is available at: https://github.com/SchmollerLab/SeelMito/releases/tag/v1.0.
