# Supplementary material for: Regulation with cell size ensures mitochondrial DNA homeostasis during cell growth
Source: Nat Struct Mol Biol. 2023 Sep 7;30(10):1549–60. doi: 10.1038/s41594-023-01091-8 (PMC10584693; doi:10.1038/s41594-023-01091-8)
Supplement: Supplementary file 1 — Supplementary Tables 1–5 [file 41594_2023_1091_MOESM1_ESM.pdf]

nature structural & molecular biology

Article https://doi.org/10.1038/s41594-023-01091-8

Regulation with cell size ensures mitochondrial DNA homeostasis during cell growth

In the format provided by the authors and unedited

Supplementary Information

Supplementary Table 1: Testing ASY39-1 (ρ^0 strain) for mtDNA by qPCR. Raw Cq-values obtained for mitochondrial (*COX2, COX3*) and nuclear DNA (*ACT1*) sequences are shown for WT (MMY116-2c), haploid microscopy strain (ASY13-1) and ρ^0 strain (ASY39-1).

Gene	WT Cq-value +/- StD	ρ^+ Cq-value +/- StD	ρ^0 Cq-value +/- StD
COX2	18.96 +/-0.19	19.30 +/- 0.15	Cq-values not measurable or higher than 36
COX3	18.53 +/- 0.30	18.73 +/- 0.15	Cq-values not measurable or higher than 34
ACT1	22.83 +/- 0.33	22.70 +/- 0.33	21.84 +/- 0.26

Supplementary Table 2: Strains used in this study.

Name	Genotype	Description	Origin	Figure
ASY004-1	*Mat α; ADE2, ABF2-linker-mCitrine-CglaTRP1*	*ABF2-mCitrine* in MMY116-2c	This study	4e, E.D.fig. 5a-b
ASY006-1	*Mat α; ADE2, MIP1-linker-mCitrine- ADH1term-CglaTRP1*	*MIP1-mCitrine* in MMY116-2c	This study	4d, E.D.fig. 5c-g
ASY007-2	*Mat a; ADE2, MIP1-linker-mCitrine:CglaTRP1, whi5Δ::kanMX6-LexApr-WHI5-ADH1term-LEU2, his3::LexA-ER-AD-TF-HIS3*	MIP1-mCitrine-Adh1term-CglaTRP1 (KCE001-2) in MS63-1	This study	E.D.fig. 5e
ASY013-1	*Mat a; mt-LacO, ADE2, TRP1, whi5Δ::KlacURA3, LexApr-WHI5-ADH1term-LEU2, his3::LexA-ER-LBD-HIS3, Pcup1-Su9-2xNEON-LacI-Pgk1-Su9-mKate2-KanMX4*	Haploid microscopy strain, Whi5-inducible	This study	2c-e; 3a-d; E.D.fig. 2b; 4a-b; 10
ASY015-1	*Mat α/a; mt-LacO, ADE2/ADE2, Δwhi5::KlacURA3/WHI5, LexApr-WHI5-ADH1term-LEU2, his3::LexA-ER-LBD-HIS3, Pcup1-Su9-2xNEON-LacI-Pgk1-Su9-mKate2-KanMX4*	Diploid microscopy strain, Whi5-inducible	This study	2b-e; E.D.fig. 2a-b; 10
ASY020-1	*Mat α/a; ADE2/ADE2, URA3/ura3, leu2/LEU2*	Diploid WT	This study	1b; 5b-c; 7a-b; E.D.fig. 7
ASY023-1	*Mat α/a; ADE2/ADE2, ura3-1/URA3, whi5Δ::kanMX6-LexApr-WHI5-ADH1term-LEU2/WHI5, his3::LexA-ER-AD-TF-HIS3/his3-11,15*	Whi5-inducible strain	This study	1b; 7b

ASY024-1	*Mat α/a; ADE2/ADE2, leu2-3/LEU2, ura3-1/URA3, mip1::CgalTRP1/MIP1*	Hemizygous *MIP1* strain	This study	5b-c; 7a-b; E.D.fig. 7
ASY025-1	*Mat α/a; ADE2/ADE2, ura3-1/URA3, whi5Δ::kanMX6-LexApr-WHI5-ADH1term-LEU2/WHI5, his3::LexA-ER-AD-TF-HIS3/his3-11,15, mip1::CgalTRP1/MIP1*	Hemizygous *MIP1* strain, Whi5-inducible	This study	7b; E.D.fig. 7 b-d
ASY033-1	*Mat α/a; ADE2/ADE2, leu2-3/LEU2, URA3/ura3-1, pif1::TRP1/PIF1*	Hemizygous *PIF1* strain	This study	5b; E.D.fig. 7a
ASY034-1	*Mat α/a; ADE2/ADE2, leu2-3/LEU2, URA3/ura3-1, rad53::TRP1/RAD53*	Hemizygous *RAD53* strain	This study	5b; E.D.fig. 7a
ASY035-1	*Mat α/a; ADE2/ADE2, leu2-3/LEU2, URA3/ura3-1, rrm3::TRP1/RRM3*	Hemizygous *RRM3* strain	This study	5b; E.D.fig. 7a
ASY039-1	*Mat α/a; ADE2, ∆whi5::KlacURA3, hiWHI5:LEU2, LexA-ER-LBD:HIS3, Pcup1-Su9-2xNEON-LacI--Pgk1-Su9-mKate2:KanMX4, TRP1, mip1::clonNAT*	*mip1∆* microscopy strain	This study	3a-d; E.D.fig. 4a-b; Supp. Table 1
ASY046-1	*Mat α/a; ADE2/ADE2, leu2-3/LEU2, ura3-1/URA3, mip1::CgalTRP1/MIP1, abf2::clonNAT/ABF2*	Double hemizygous *MIP1 ABF2* strain	This study	7a-b; E.D.fig. 7 b-d
ASY049-2	*Mat α/a; whi5Δ::kanMX6-LexApr-WHI5-ADH1term-LEU2, his3::LexA-ER-AD-TF-HIS3, mip1::CgalTRP1/MIP1, abf2::clonNAT/ABF2*	Double hemizygous *MIP1 ABF2* strain; Whi5-inducible	This study	7b; E.D.fig. 7 b-d
ASY051-2	*Mat α; ADE2, ura3::URA3/ABF2*	Haploid WT with additional copy of *ABF2*	This study	7d-e; E.D.fig. 7e-g
ASY052-5	*Mat a; ADE2, whi5Δ::kanMX6-LexApr-WHI5-ADH1term-LEU2, his3::LexA-ER-AD-TF-HIS3, ura3::URA3/ABF2*	Haploid Whi5-inducible strain with additional copy of *ABF2*	This study	7e; E.D.fig. 7e-g
ASY057-3	*Mat α; ADE2, trp1::TRP1/MIP1*	Haploid WT with additional copy of *MIP1*	This study	7d-e; E.D.fig. 7e-g
ASY058-1	*Mat a; ADE2, whi5Δ::kanMX6-LexApr-WHI5-ADH1term-LEU2, his3::LexA-ER-AD-TF-HIS3, trp1::TRP1/MIP1*	Haploid Whi5-inducible strain with additional copy of *MIP1*	This study	7e; E.D.fig. 7e-g

ASY059-2	*Mat α; ADE2, ura3::URA3/ABF2, trp1::TRP1/MIP1*	Haploid WT with additional *ABF2* and *MIP1* copies	This study	7d-e; E.D.fig. 7e-g
ASY060-1	*Mat a; ADE2, whi5Δ::kanMX6-LexApr-WHI5-ADH1term-LEU2, his3::LexA-ER-AD-TF-HIS3, ura3::URA3/ABF2, trp1::TRP1/MIP1*	Haploid Whi5-inducible strain with additional copies of *ABF2* and *MIP1*	This study	7e; E.D.fig. 7e-g
JE611-c	*Mat α, cln1Δ, cln2Δ, cln3::leu2, lexOPr-Cln1-Leu2, ADE2, his3::cyc1-Pr-lexO TF-his3, TRP, URA*	Cln1-inducible strain	Jennifer Ewald, Skotheim lab	1c; E.D.fig. 1e-f
KSY244-1	*Mat α/a; ADE2/ADE2, leu2-3/LEU2, URA3/ura3-1, abf2::TRP1/ABF2*	Hemizygous *ABF2* strain	This study	5b,c; E.D.fig. 7a-d
KSY245-1	*Mat α/a; ADE2/ADE2, ura3-1/URA3, whi5Δ::kanMX6-LexApr-WHI5-ADH1term-LEU2/WHI5, his3::LexA-ER-AD-TF-HIS3/his3-11,15, abf2::CgalTRP1/ABF2*	Hemizygous *ABF2* strain; Whi5-inducible	This study	7b; E.D.fig. 7 b-d
MMY116-2c	*Mat α; ADE2*	Haploid WT strain	Skotheim lab stock	1b, 2f-h; 7d-e; E.D.fig. 1, 3, 5, 7
MS63-1	*Mat a; ADE2, whi5Δ::kanMX6-LexAprom-WHI5-ADH1term-LEU2, his3::LexA-ER-AD-TF-HIS3*	Haploid Whi5-inducible strain	Matthew Swaffer, Skotheim lab	1b, 2f-h, 4a, 7e; E.D.fig. 1, 2b, 3, 5e, 6, 7
KCY005-1	*Mat α/a; ADE2/ADE2, whi5Δ::CglaTRP1/whi5Δ::kanMX6-LexApromWHI5-ADH1term-LEU2, his3/his3::LexA-ERAD-TF-HIS3*	Diploid Whi5-inducible strain	Kora-Lee Claude, Schmoller lab	E.D.fig. 6a-c
KSY246	*Mat α/a; ADE2/ADE2, leu2-3/LEU2, URA3/ura3-1, hmi1::TRP1/HMI1*	Hemizygous *HMI1* strain	This study	5b; E.D.fig. 7a
KSY253	*Mat α/a; ADE2/ADE2, leu2-3/LEU2, URA3/ura3-1, rpo41::TRP1/RPO41*	Hemizygous *RPO41* strain	This study	5b; E.D.fig. 7a
KSY254	*Mat α/a; ADE2/ADE2, leu2-3/LEU2, URA3/ura3-1, mtf1::TRP1/MTF1*	Hemizygous *MTF1* strain	This study	5b; 7a
KSY255	*Mat α/a; ADE2/ADE2, leu2-3/LEU2, URA3/ura3-1, mhr1::TRP1/MHR1*	Hemizygous *MHR1* strain	This study	5b; E.D.fig. 7a

KSY256	*Mat α/a; ADE2/ADE2, leu2-3/LEU2, URA3/ura3-1, mgm101::TRP1/MGM101*	Hemizygous *MGM101* strain	This study	5b; E.D.fig. 7a
KSY257	*Mat α/a; ADE2/ADE2, leu2-3/LEU2, URA3/ura3-1, rim1::TRP1/RIM1*	Hemizygous *RIM1* strain	This study	5b; E.D.fig. 7a
KSY299-1,2	*Mat α; ADE2, mip1::MIP1-ADH1term(short)-cglaTRP1*	*MIP1* terminator replaced with *MIP1-Adh1term(short)* in MMY116-2C	This study	E.D.fig. 5f-g
KSY300-1,2	*Mat α; ADE2, mip1::MIP1-ADH1term(long)-cglaTRP1*	*MIP1* terminator replaced with *MIP1-ADH1term*(long) in MMY116-2C	This study	E.D.fig. 5f-g
KSY301-1,2	*Mat α; ADE2, mip1::MIP1-mCitr-MIP1term-cglaTRP1*	*MIP1-mCitrine-MIP1term* in MMY116-2C	This study	E.D.fig. 5f-g
yFT006	*Mat α/a; mt-LacO, ADE2/ADE2, TRP1/TRP1, HO::Pcup1-Su9-3xNeonGreen-LacI-PGK1-Su9-mKate-kanMX6/HO::Pcup1-Su9-3xNeonGreen-LacI-PGK1-Su9-mKate-kanMX6*	WT microscopy strain for *mic60Δ* microscopy	This study	3e-g; E.D.fig. 4d-h
yFT023	*Mat α/a; mt-LacO, ADE2/ADE2, TRP1/TRP1, HO::Pcup1-Su9-3xNeonGreen-LacI-PGK1-Su9-mKate-kanMX6/HO::Pcup1-Su9-3xNeonGreen-LacI-PGK1-Su9-mKate-kanMX6, Δmic60::Nourseothricin/Δmic60::Nourseothricin*	*mic60Δ* microscopy strain	This study	3e-g; E.D.fig. 4d-h

Supplementary Table 3: Plasmids constructed in this study. *MIP1* and *ABF2* promoters correspond to 1000 bp upstream of the start codon. *MIP1* terminator corresponds to 271 bp downstream of coding *MIP1*. *ABF2* terminator corresponds to 288 bp downstream of coding *ABF2*.

**Plasmid**	**Description**
ASE001-5	HO-homology-CuPr-SU9-2xmNeon-LacI
ASE002-2	pRS404-Mip1Pr-Mip1-Mip1term
ASE003-1	pRS406-Abf2Pr-Abf2-Abf2term

Supplementary Table 4: qPCR primers used in this study

Gene	qPCR primer direction	qPCR primer sequence (5'-3')
ABF2	forward	AACCAGCAGGACCCTTCATT
	reverse	AGTTGAGAGGGTAGCGAGCA
ACT1	forward	CACCCTGTTCTTTTGACTGA
	reverse	CGTAGAAGGCTGGAACGTTG
COX2	forward	GTTGATGCTACTCCTGGTAGATT
	reverse	TTGCATGACCTGTCCCACAC
COX3	forward	TTGAAGCTGTACAACCTACC
	reverse	CCTGCGATTAAGGCATGATG
MIP1	forward	CCATCACAAGCAAGAACGGC
	reverse	GTCCCTTTCCAGCTCAACCA
MRX6	forward	CATCCGACGTGGTGCTCTTA
	reverse	TCTCATCTCTCCCTCCACCC
MTF1	forward	TTGCTAATGTGACGGGGGAG
	reverse	CTGTTGTGCTTGGCATCCAT
PIM1	forward	ACCCTACATTGGCGCTTTCA
	reverse	AGTGCCCGTCTTTTCGTCTT
RDN18	forward	AACTCACCAGGTCCAGACACAATAAGG
	reverse	AAGGTCTCGTTCGTTATCGCAATTAAGC
RPO41	forward	TCTGGGTAGAACACCGTGGA
	reverse	TTCGTCTTGTGCACCTGGAA

Supplementary Table 5: Terminator sequences used in Extended Data figure 4.

	Sequence
ADH1 terminator (short)	GCGAATTTCTTATGATTTATGATTTTTATTATTAAATAAGTTAT AAAAAAAATAAGTGTATACAAATTTTAAAGTGACTCTTAGGTT TTAAAACGAAAATTCTTATTCTTGAGTAACTCTTTCCTGTAGG TCAGGTTGCTTTCTCAGGTATAGCATGAGGTCGCTCTTATTG ACCACACCTCTACCGG
ADH1 terminator (long)	GGCGCGCCACTTCTAAATAAGCGAATTTCTTATGATTTATGAT TTTTATTATTAAATAAGTTATAAAAAAAATAAGTGTATACAAATT TTAAAGTGACTCTTAGGTTTTAAAACGAAAATTCTTATTCTTGA GTAACTCTTTCCTGTAGGTCAGGTTGCTTTCTCAGGTATAGCA TGAGGTCGCTCTTATTGACCACACCTCTACCGG
MIP1 terminator sequence	GCAAAATCTGCATTAGCTTTCGCATTTGTATTTATATATACAGC ACATTATAATATTATTTTTTTATTTATTTCTACTTACTTTTTTTCG CCAAATGAGAAAACAAAAGTAAGTTGCCCCTATTAGAAAAAGA GTTAACATTAGGAAACGGGGACAAAACAAAAGGTAGAATTCA AAATAGGAATTCAAAACCCAAATTATCTGCATCTTCTCGTTAG CTAAGATTTTATAGATCATTGCAGGCTCGATAAATTTTACATG GTAACGAAAC
